# Supplementary figures and images for: Structural and mechanistic insights into ribosomal ITS2 RNA processing by nuclease-kinase machinery
Source: eLife. 2024 Jan 5;12:RP86847. doi: 10.7554/eLife.86847 (PMC10942766; doi:10.7554/eLife.86847)

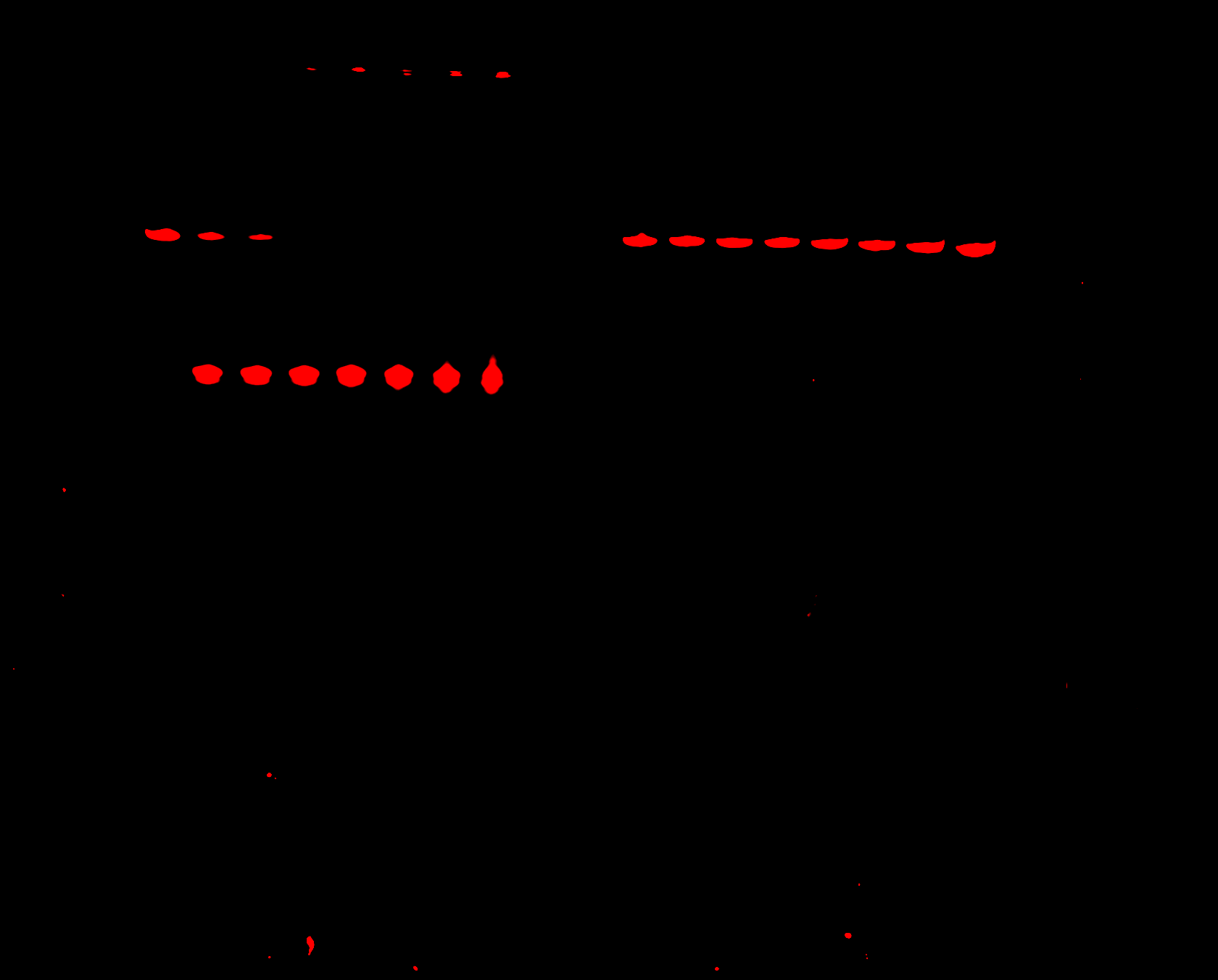

Supplement: Figure 1—source data 1. [file elife-86847-fig1-data1.zip › Figure 1-source data 1-1.tif]

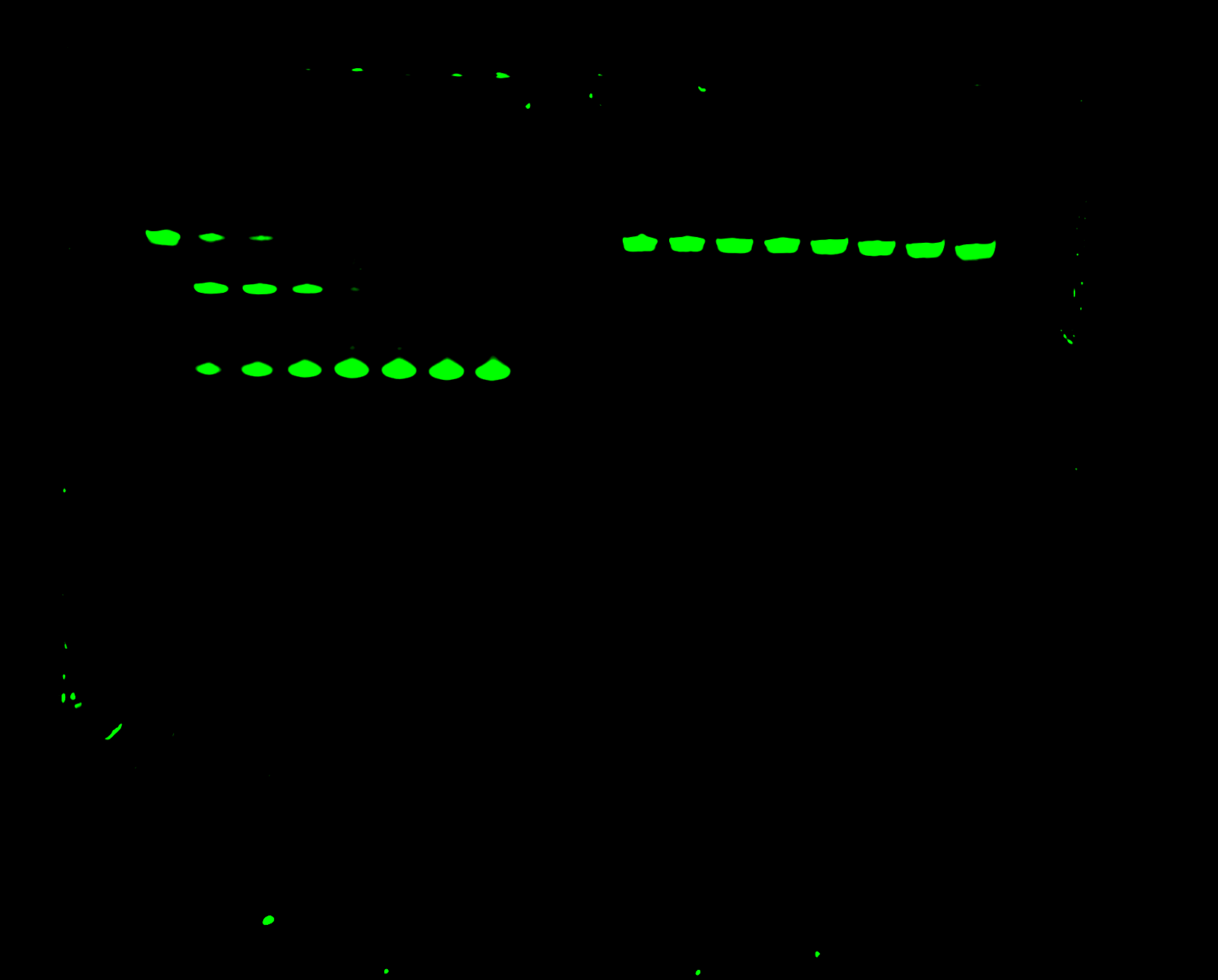

Supplement: Figure 1—source data 1. [file elife-86847-fig1-data1.zip › Figure 1-source data 1-2.tif]

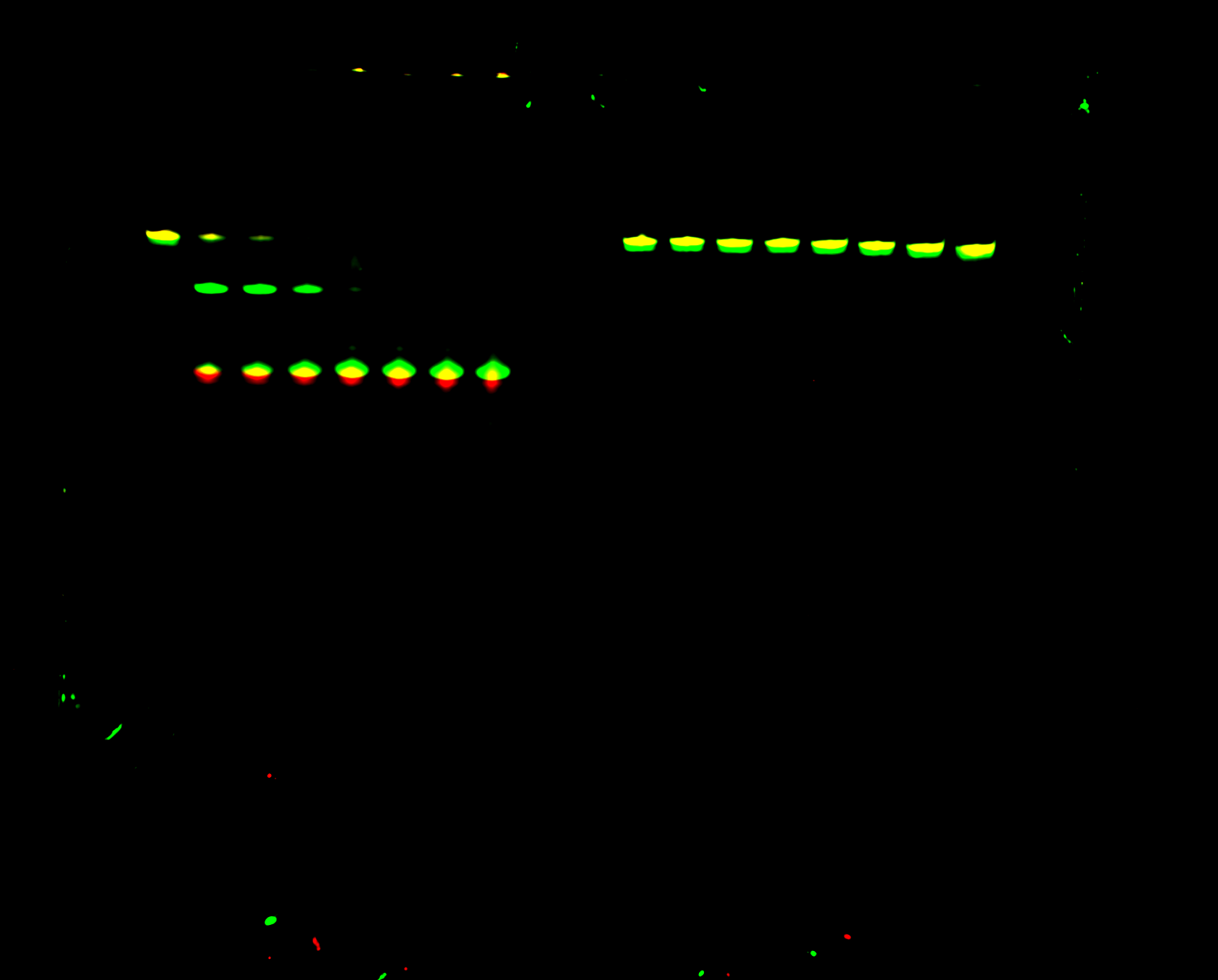

Supplement: Figure 1—source data 1. [file elife-86847-fig1-data1.zip › Figure 1-source data 1-3.tif]

**Figure 1B**

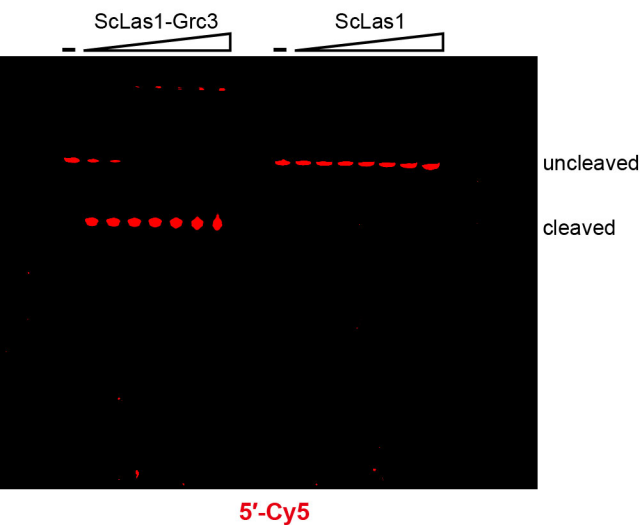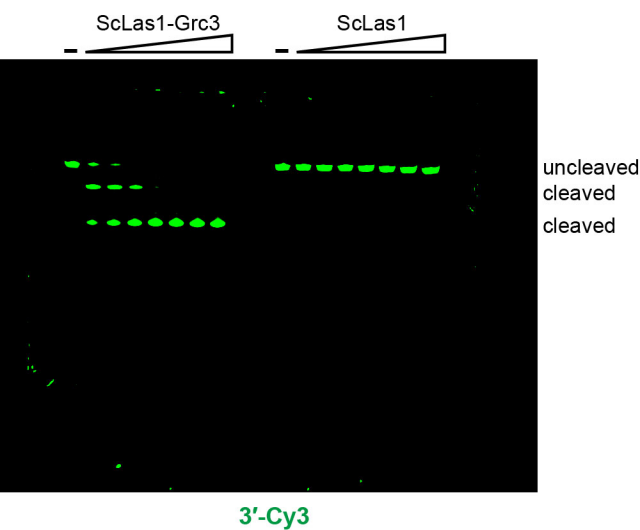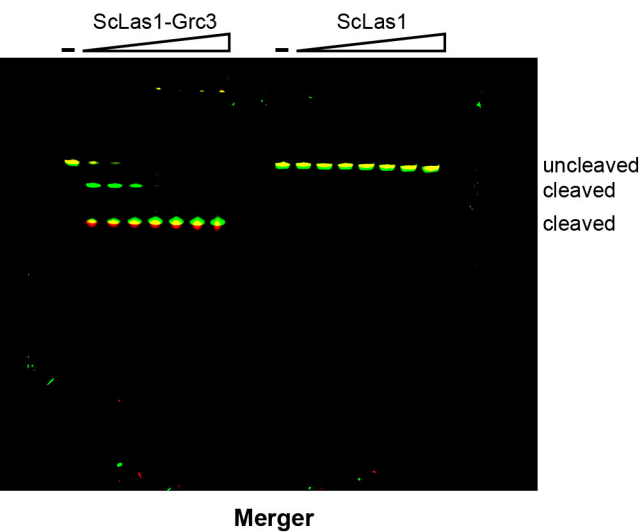

Supplement: Figure 1—source data 2. [file elife-86847-fig1-data2.zip › Figure 1-Source data 2.pdf]

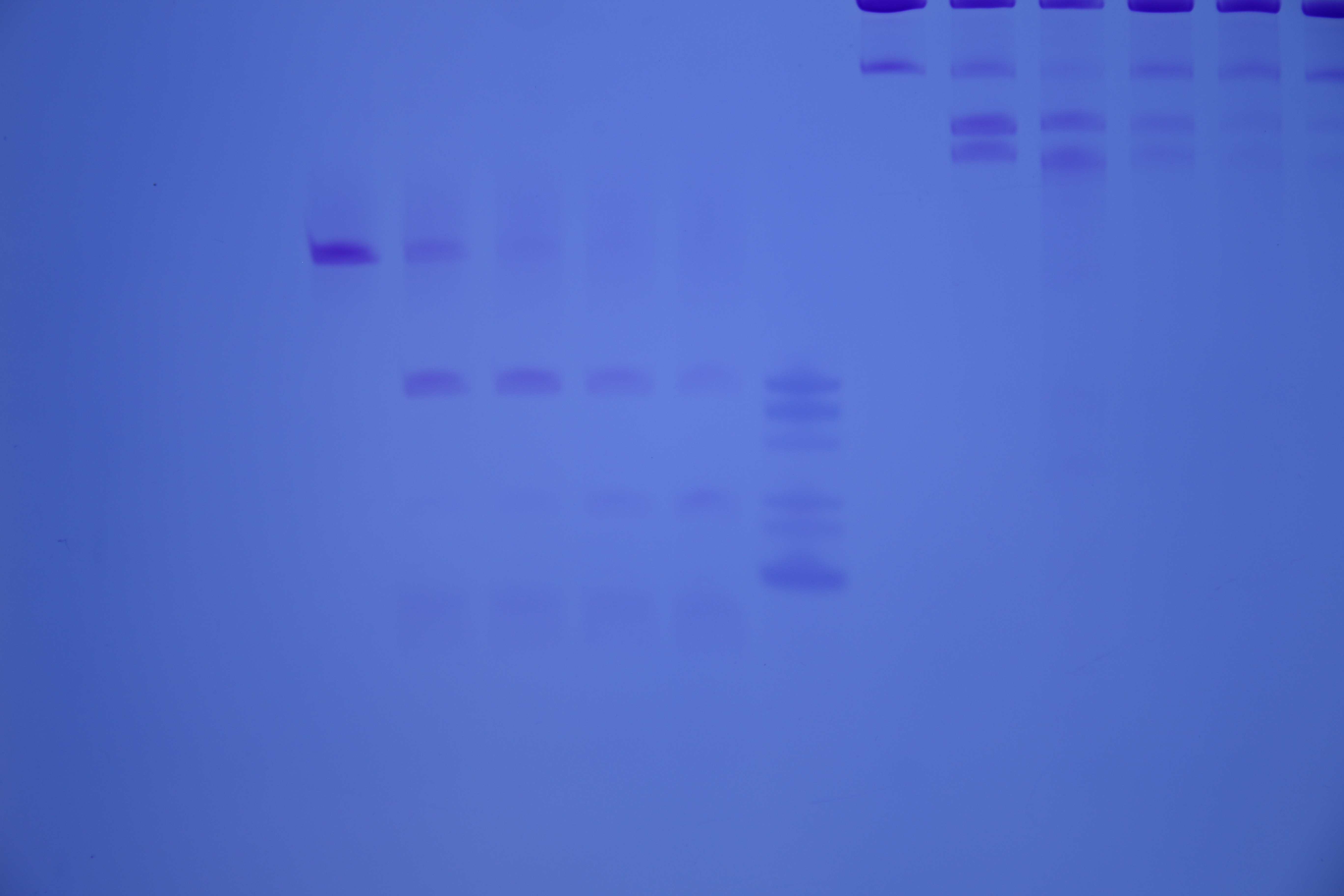

Supplement: Figure 1—source data 3. [file elife-86847-fig1-data3.zip › Figure 1-source data 3.JPG]

**Figure 1C**

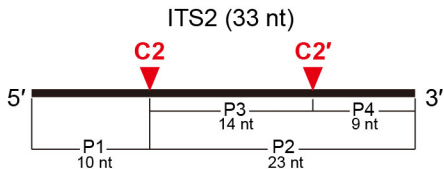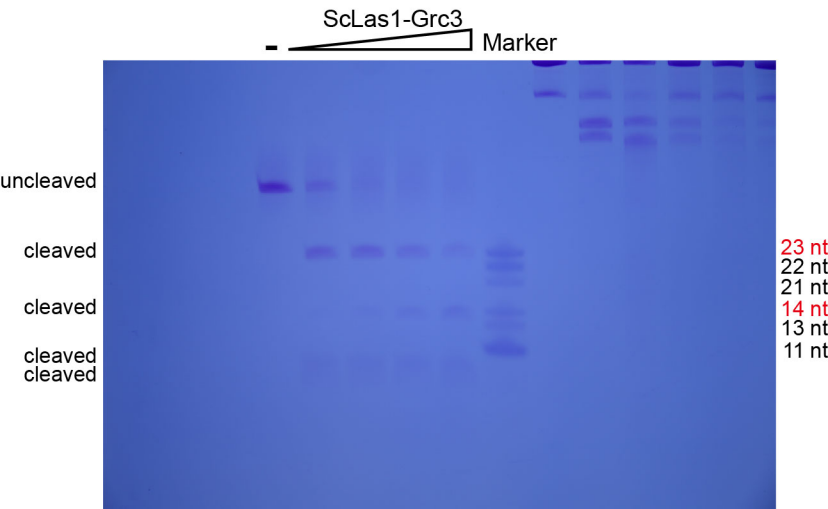

Supplement: Figure 1—source data 4. [file elife-86847-fig1-data4.zip › Figure 1-source data 4.pdf]

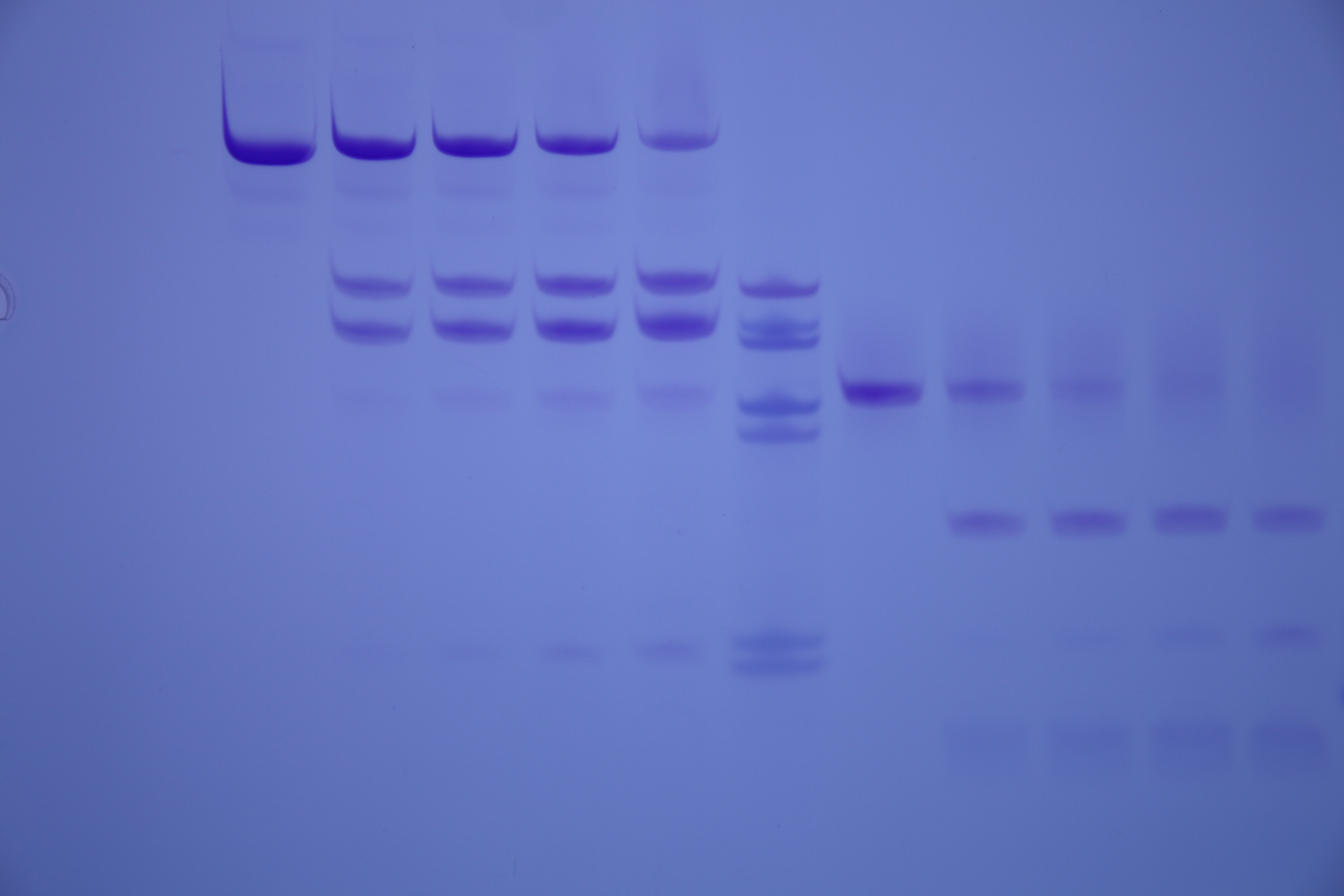

Supplement: Figure 1—source data 5. [file elife-86847-fig1-data5.zip › Figure 1-source data 5.JPG]

**Figure 1E**

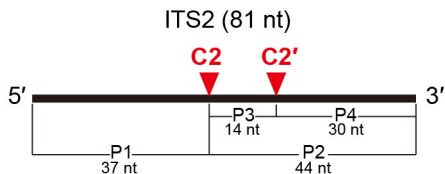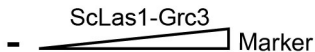

uncleaved

cleaved

cleaved

cleaved

cleaved

44 nt (P2)

37 nt (P1)

36 nt

30 nt (P4)

28 nt

14 nt (P3)

13 nt

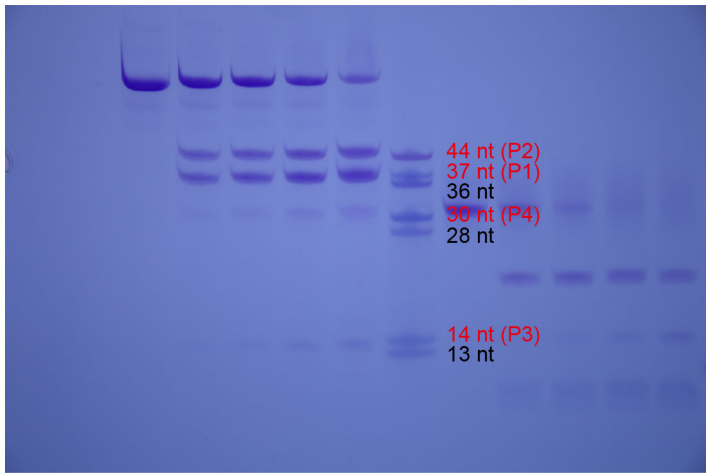

Supplement: Figure 1—source data 6. [file elife-86847-fig1-data6.zip › Figure 1-source data 6.pdf]

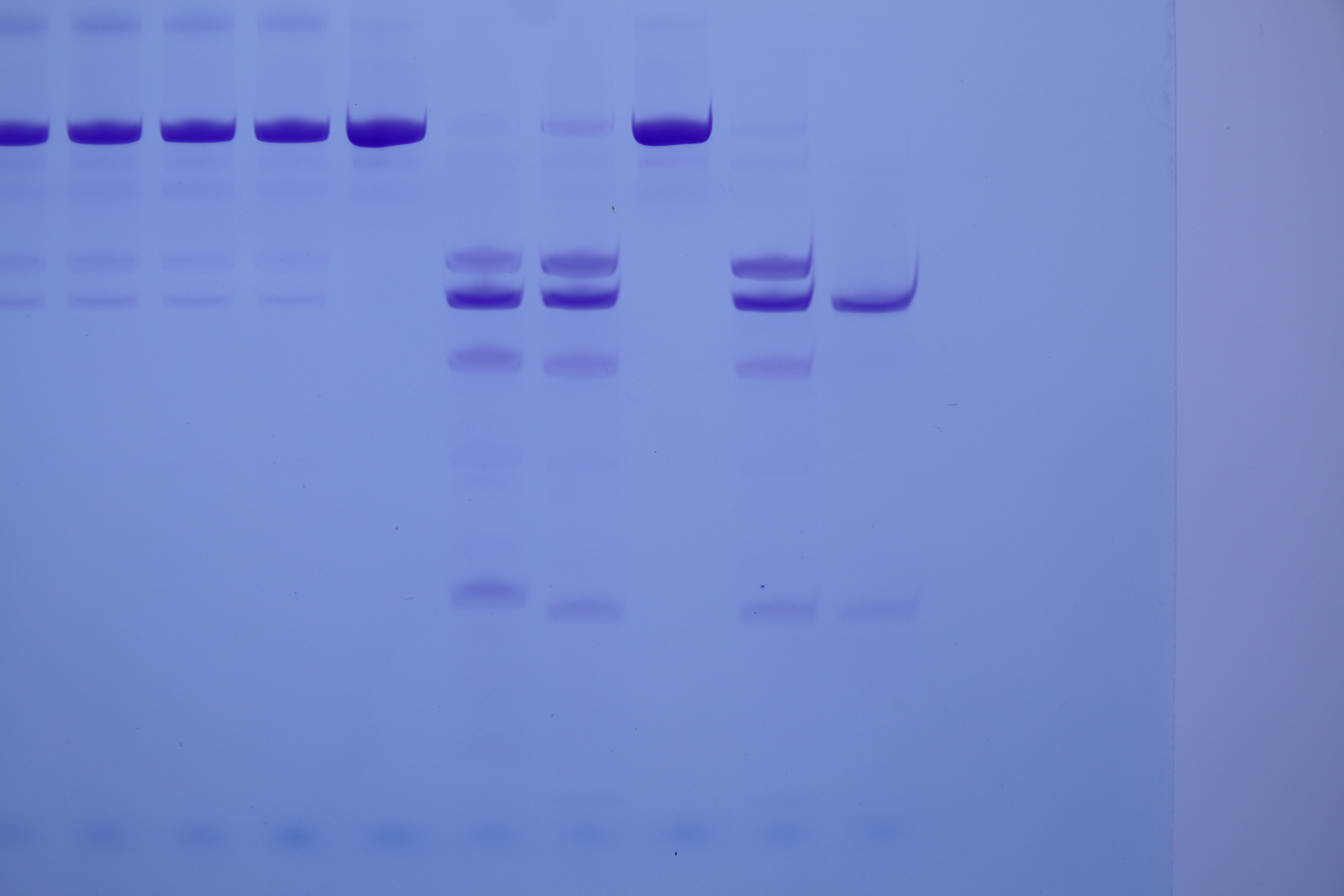

Supplement: Figure 1—source data 7. [file elife-86847-fig1-data7.zip › Figure 1-source data 7.JPG]

**Figure 1G**

|             |   |   |   |
|-------------|---|---|---|
| ITS2        | + | + | + |
| ScLas1-Grc3 | - | + | + |
| ATP         | - | - | + |

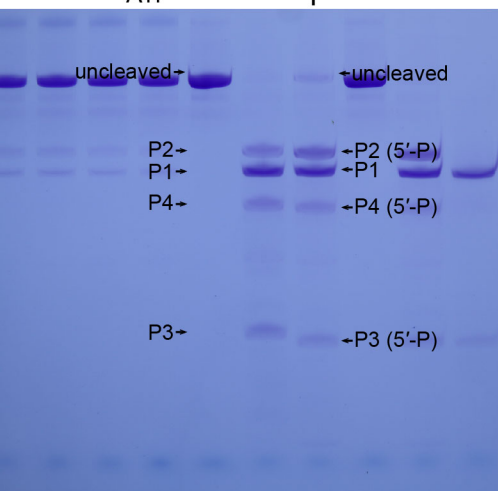

**Figure 1H**

|             |   |   |   |
|-------------|---|---|---|
| ScRat1-Rai1 | - | - | + |
| ITS2        | + | + | + |
| ScLas1-Grc3 | - | + | + |
| ATP         | - | + | + |

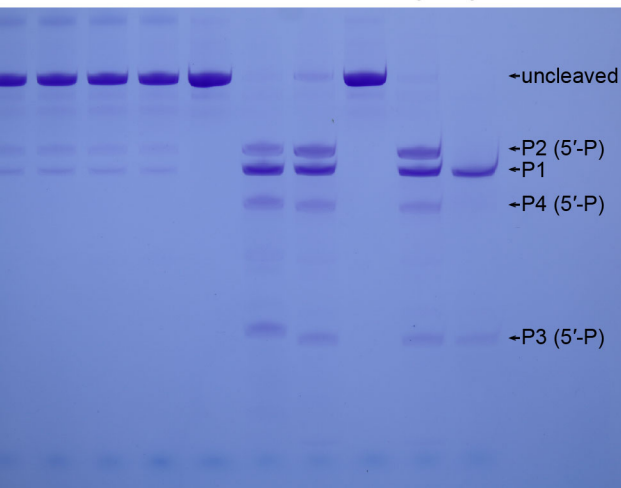

Supplement: Figure 1—source data 8. [file elife-86847-fig1-data8.zip › Figure 1-source data 8.pdf]

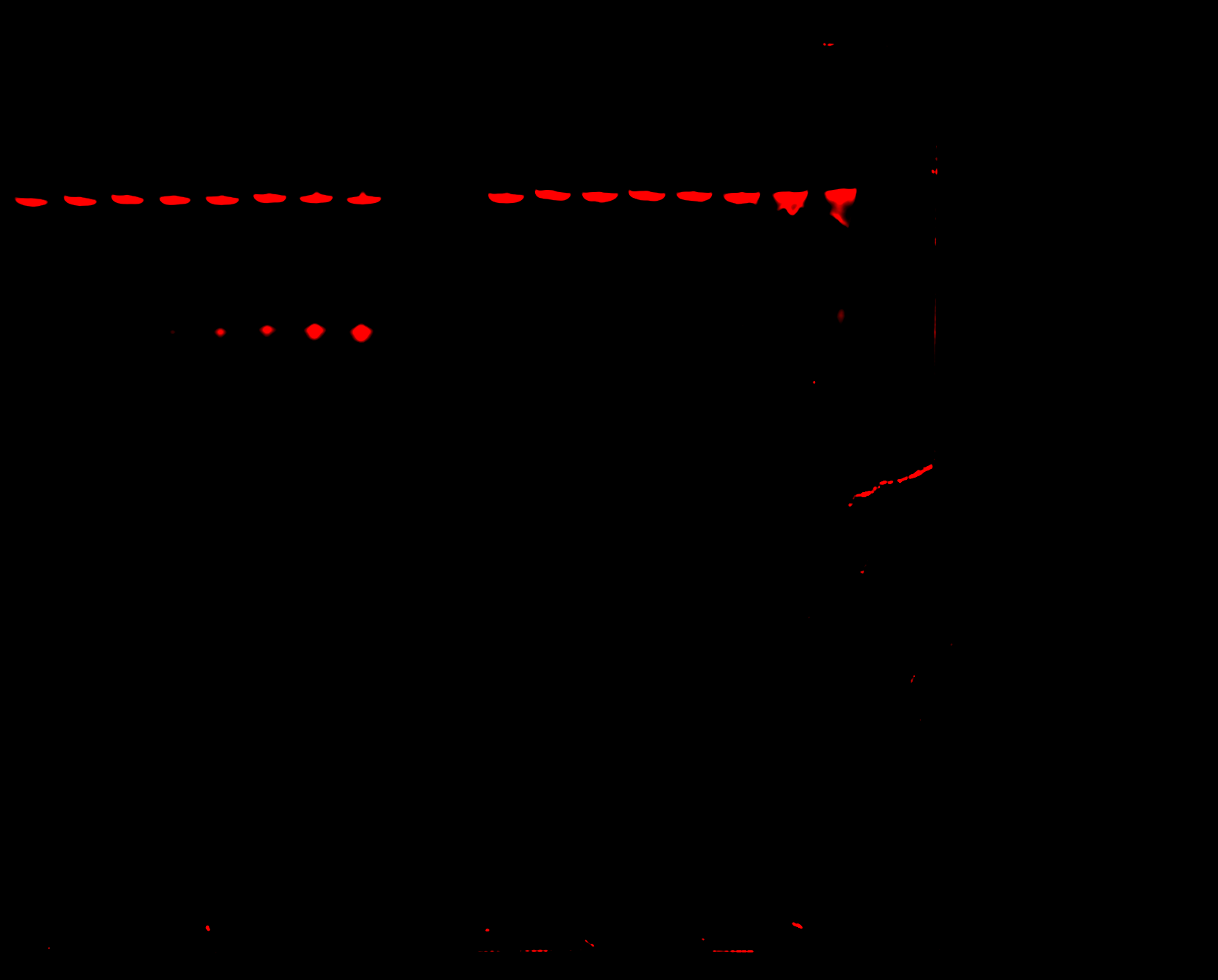

Supplement: Figure 1—figure supplement 1—source data 1. [file elife-86847-fig1-figsupp1-data1.zip › Figure 1-figure supplement 1-source data 1-1.tif]

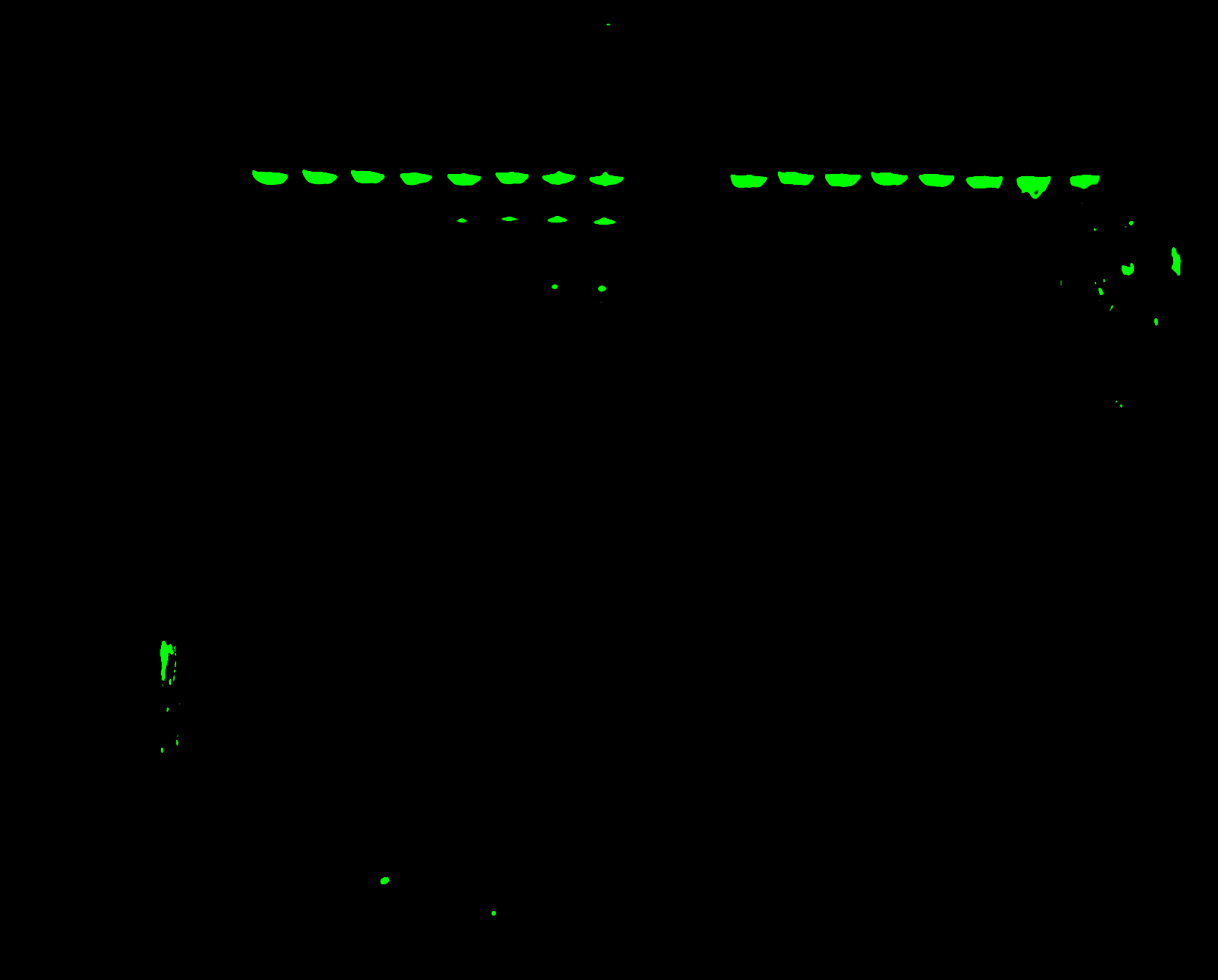

Supplement: Figure 1—figure supplement 1—source data 1. [file elife-86847-fig1-figsupp1-data1.zip › Figure 1-figure supplement 1-source data 1-2.tif]

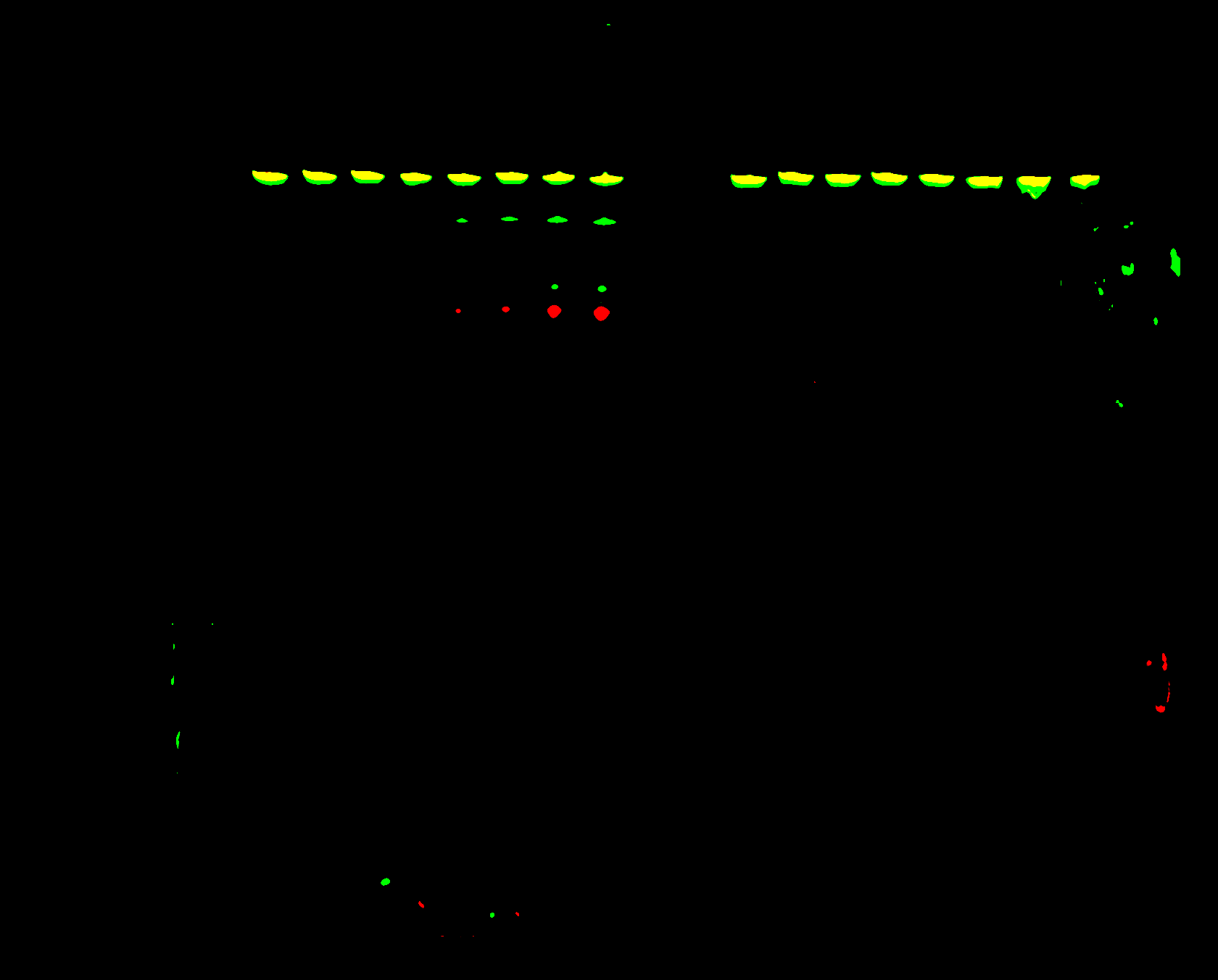

Supplement: Figure 1—figure supplement 1—source data 1. [file elife-86847-fig1-figsupp1-data1.zip › Figure 1-figure supplement 1-source data 1-3.tif]

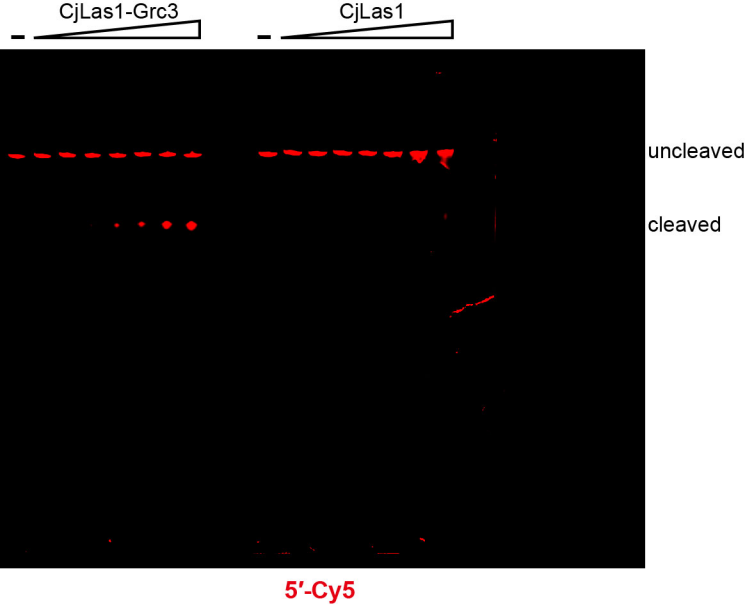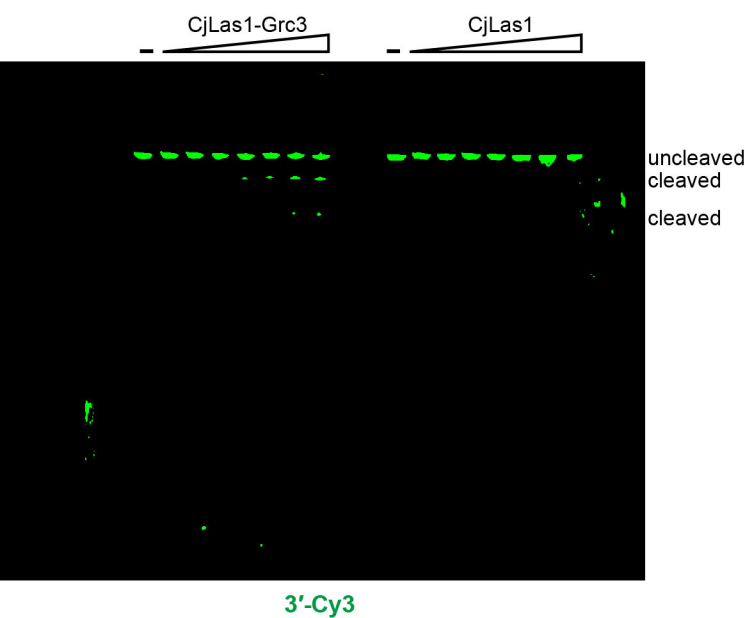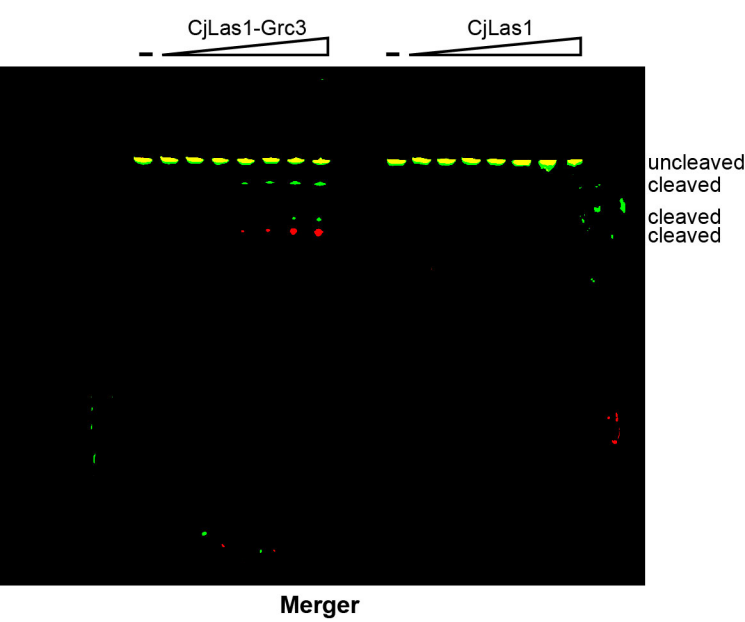

Supplement: Figure 1—figure supplement 1—source data 2. [file elife-86847-fig1-figsupp1-data2.zip › Figure 1-figure supplement 1-source data 2.pdf]

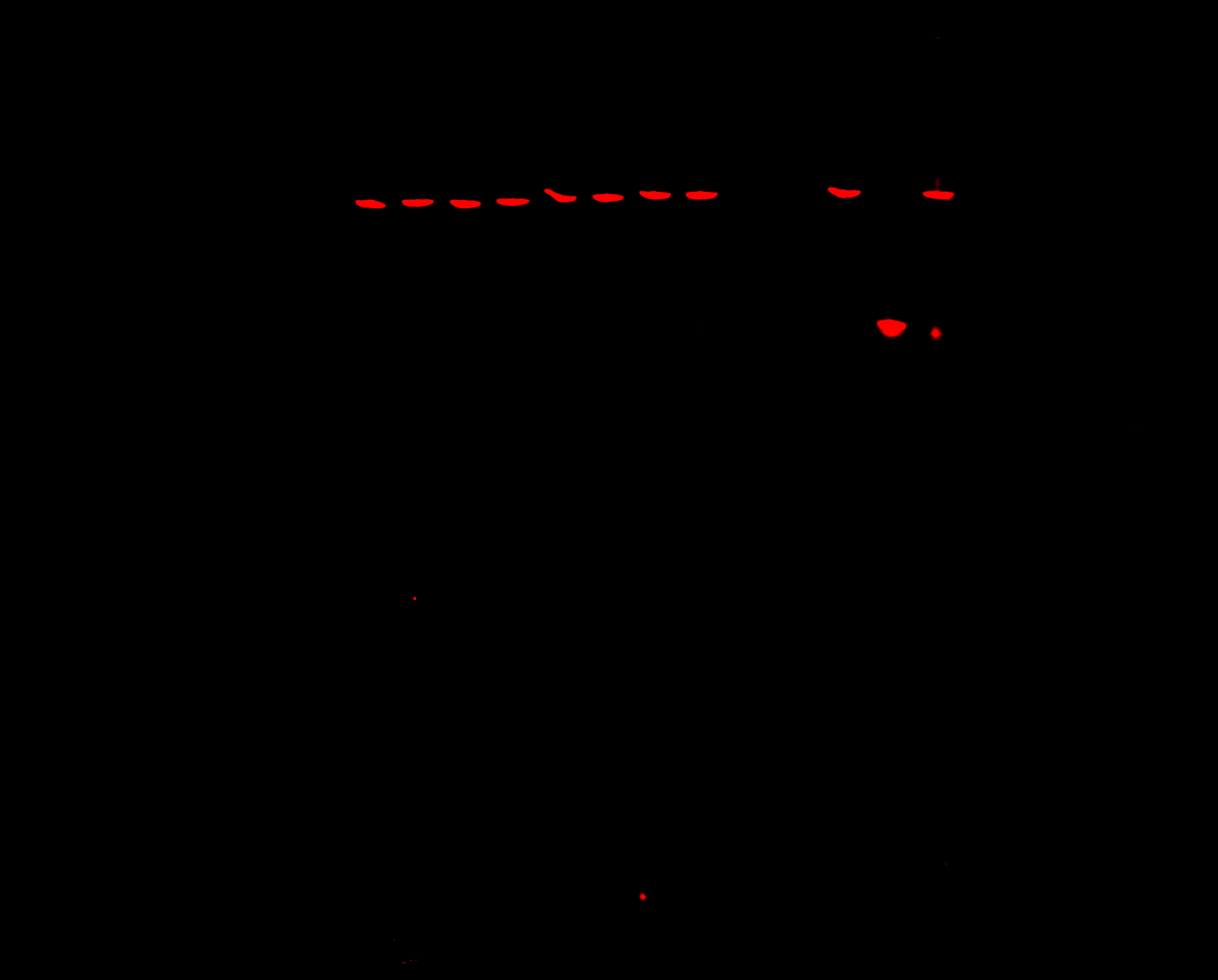

Supplement: Figure 1—figure supplement 2—source data 1. [file elife-86847-fig1-figsupp2-data1.zip › Figure 1-figure supplement 2-source data 1-1.tif]

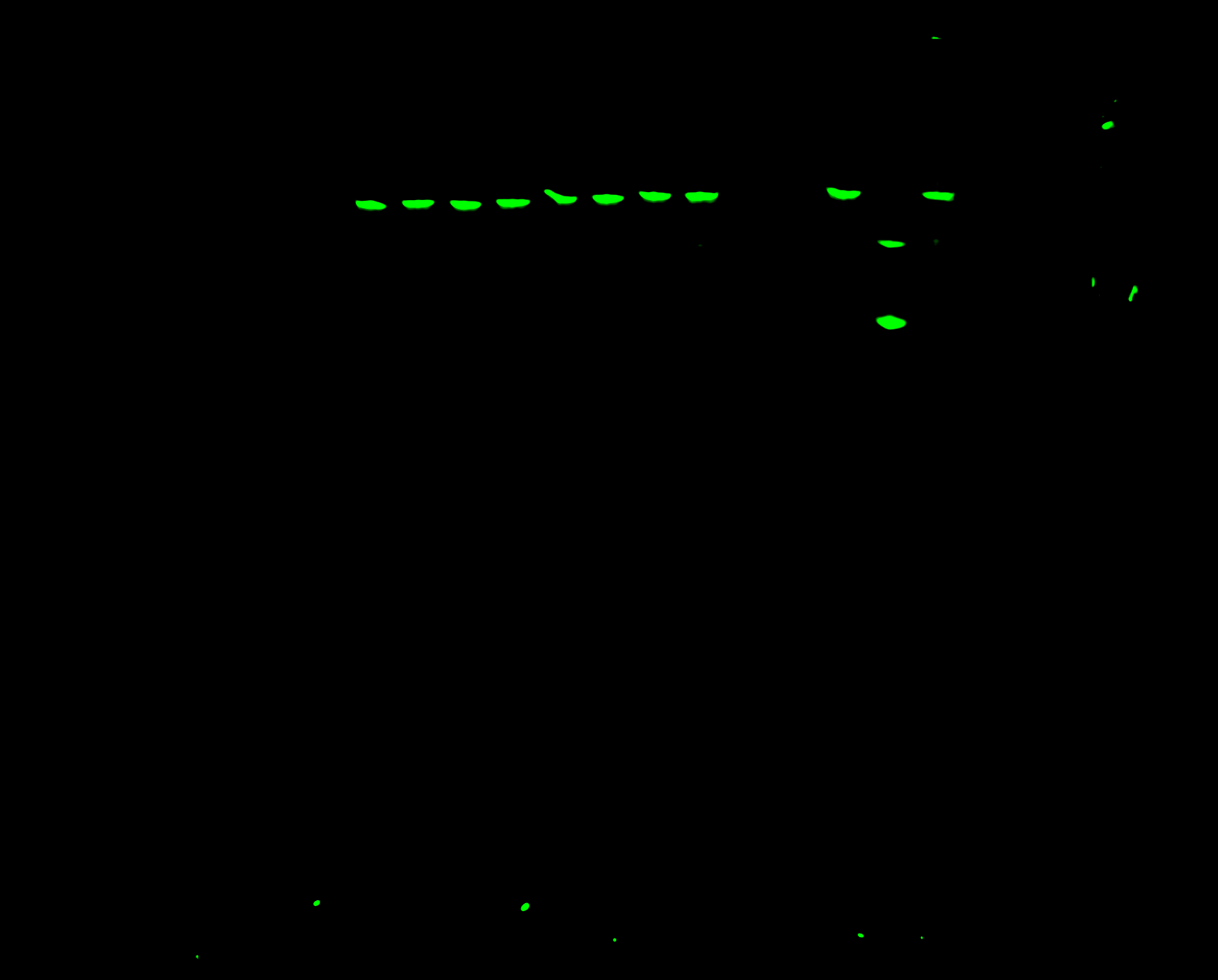

Supplement: Figure 1—figure supplement 2—source data 1. [file elife-86847-fig1-figsupp2-data1.zip › Figure 1-figure supplement 2-source data 1-2.tif]

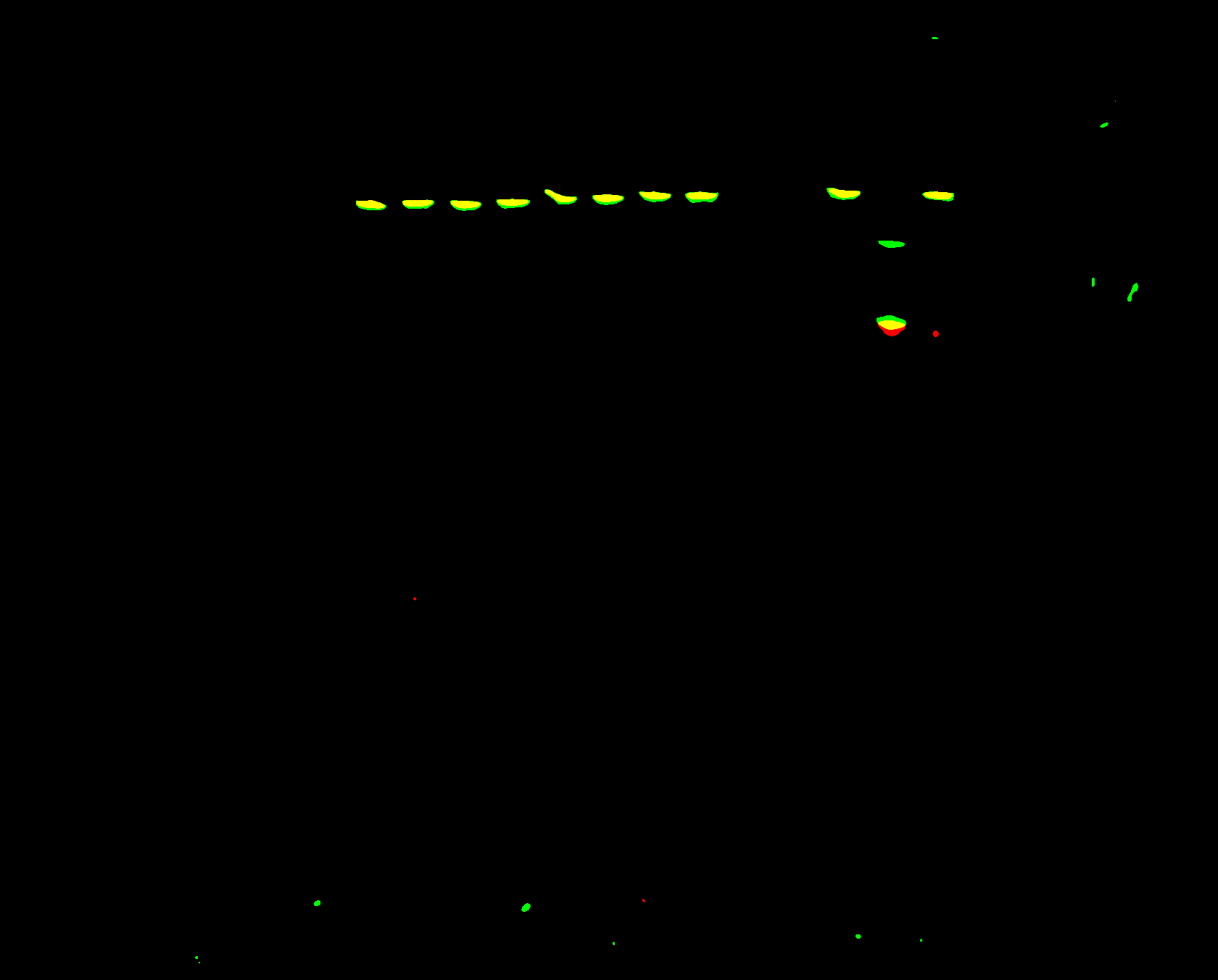

Supplement: Figure 1—figure supplement 2—source data 1. [file elife-86847-fig1-figsupp2-data1.zip › Figure 1-figure supplement 2-source data 1-3.tif]

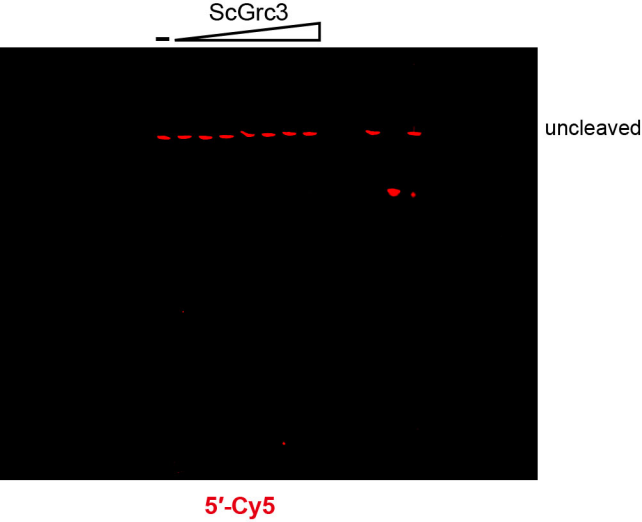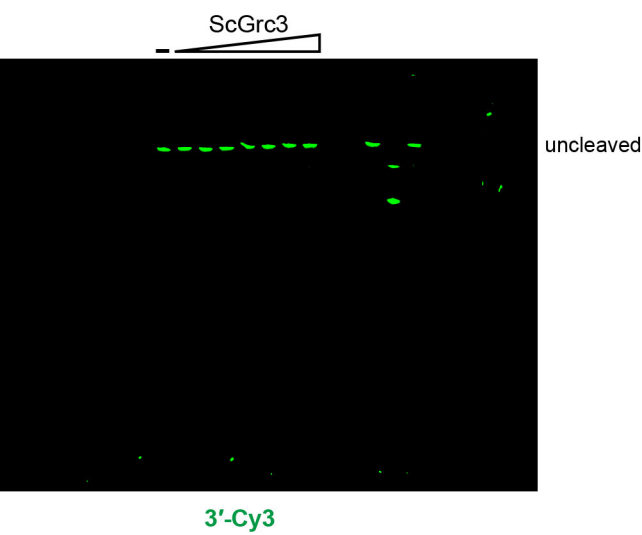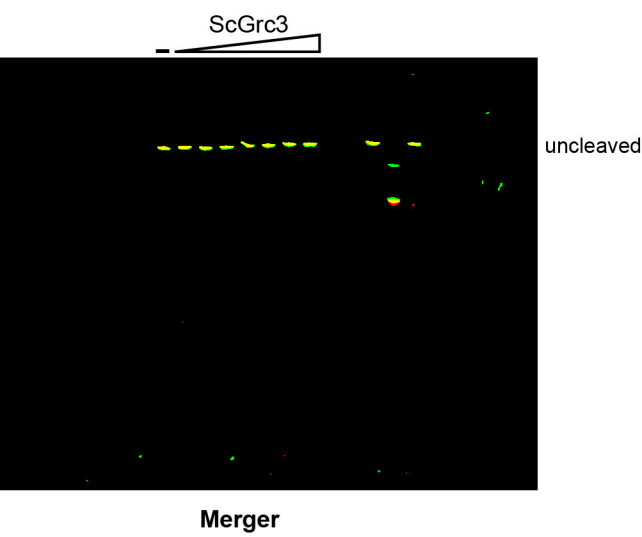

Supplement: Figure 1—figure supplement 2—source data 2. [file elife-86847-fig1-figsupp2-data2.zip › Figure 1-figure supplement 2-source data 2.pdf]

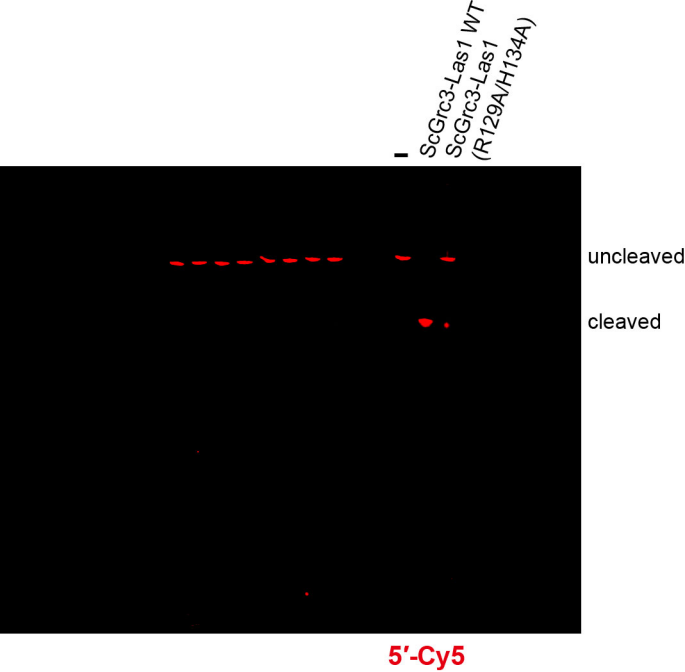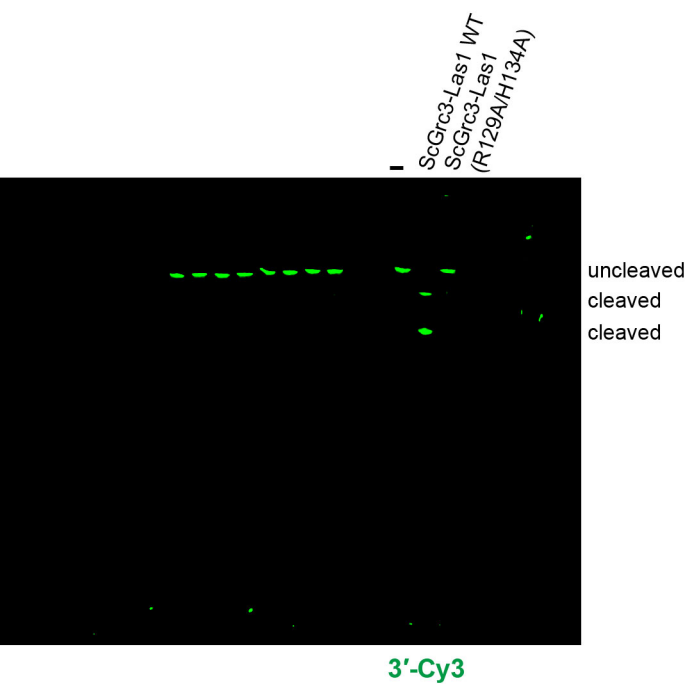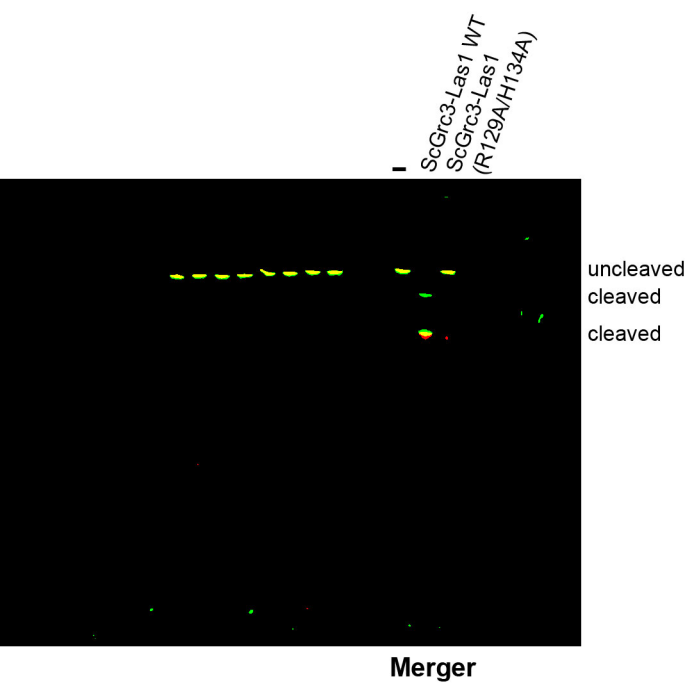

Supplement: Figure 1—figure supplement 3—source data 2. [file elife-86847-fig1-figsupp3-data2.zip › Figure 1-figure supplement 3-source data 2.pdf]

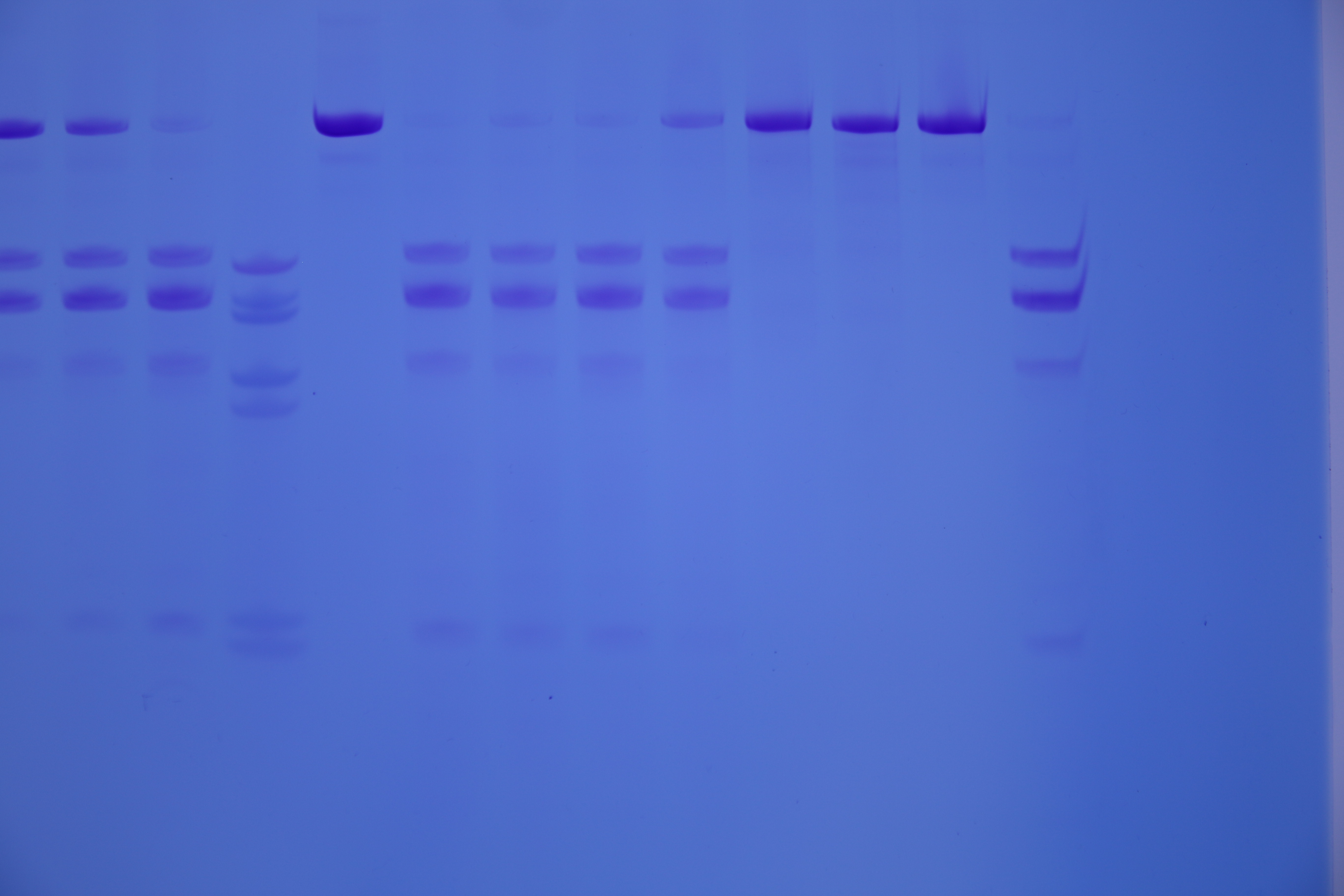

Supplement: Figure 1—figure supplement 4—source data 1. [file elife-86847-fig1-figsupp4-data1.zip › Figure 1-figure supplement 4-source data 1.JPG]

ITS2 —  $\text{Ca}^{2+}$   $\text{Mg}^{2+}$   $\text{Mn}^{2+}$   $\text{Co}^{2+}$   $\text{Zn}^{2+}$   $\text{Ni}^{2+}$  EDTA

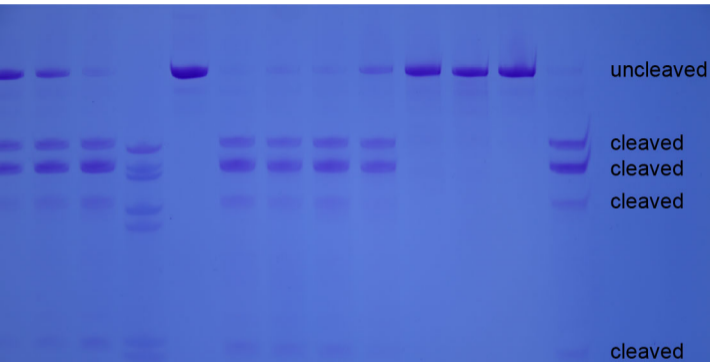

Supplement: Figure 1—figure supplement 4—source data 2. [file elife-86847-fig1-figsupp4-data2.zip › Figure 1-figure supplement 4-source data 2.pdf]

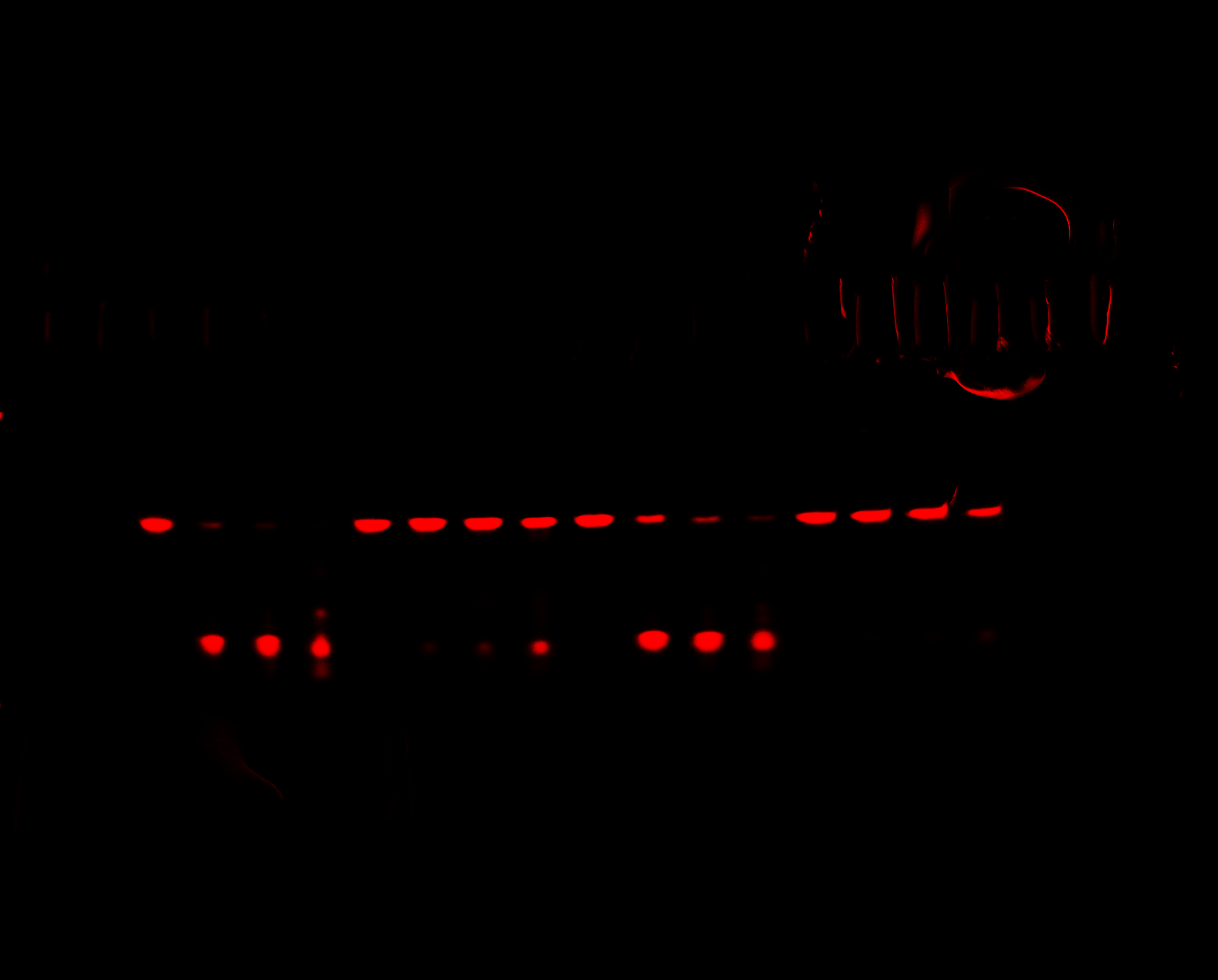

Supplement: Figure 4—source data 1. [file elife-86847-fig4-data1.zip › Figure 4-source data 1-1.tif]

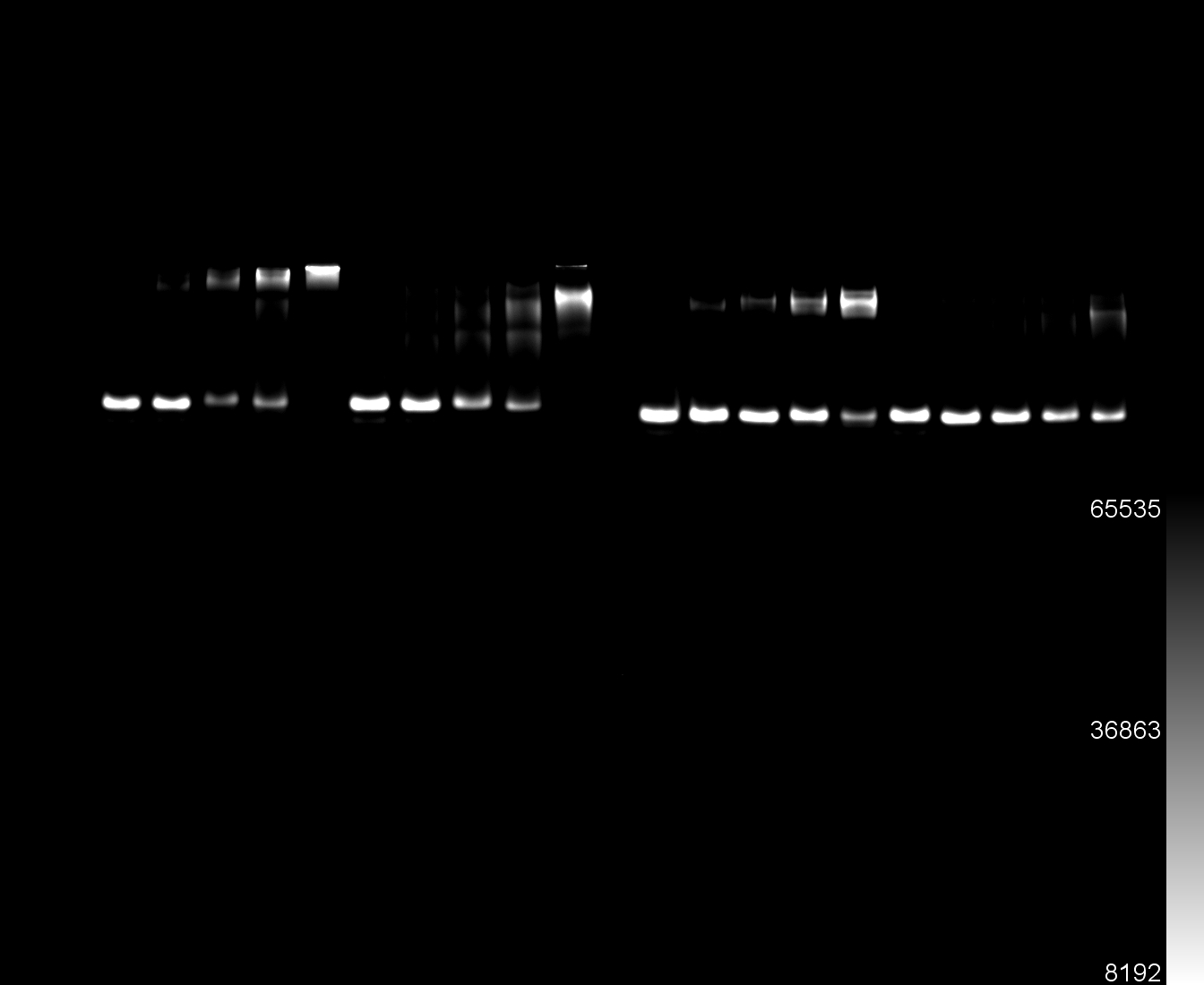

Supplement: Figure 4—source data 1. [file elife-86847-fig4-data1.zip › Figure 4-source data 1-2.jpg]

**Figure 4C**

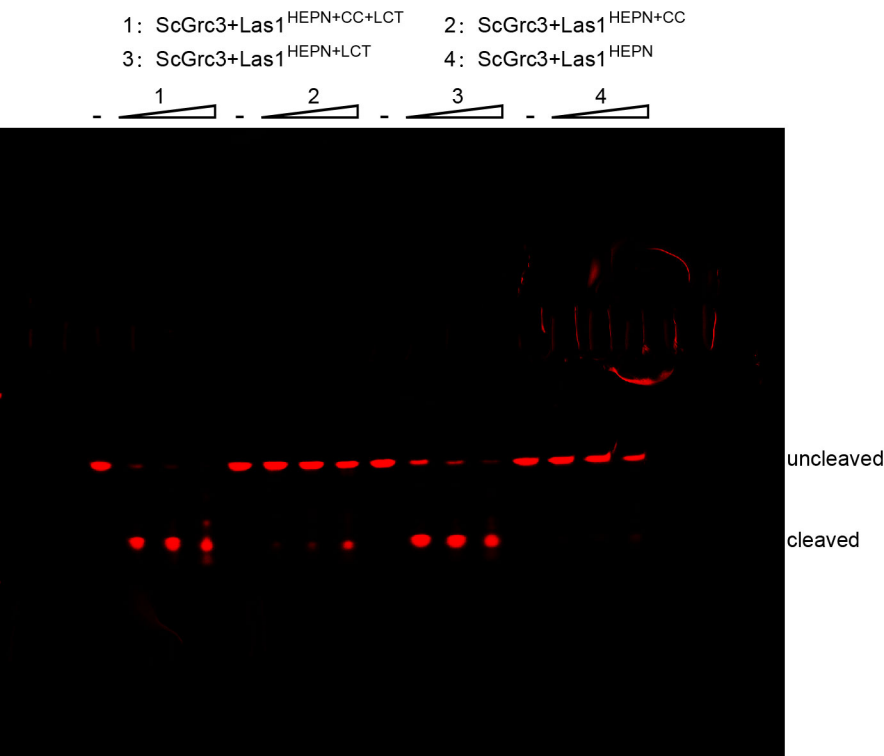

**Figure 4D**

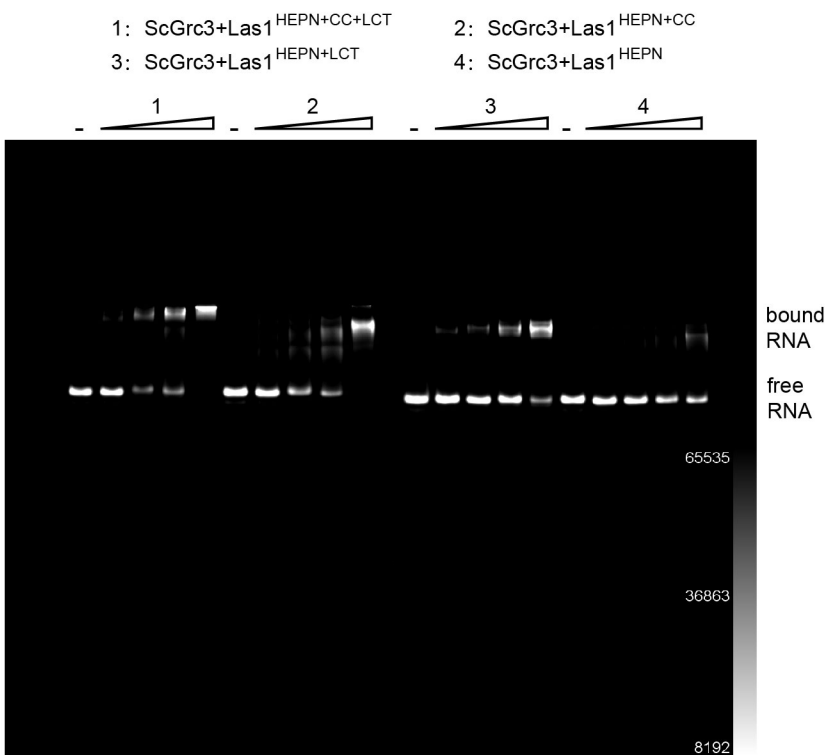

Supplement: Figure 4—source data 2. [file elife-86847-fig4-data2.zip › Figure 4-source data 2.pdf]

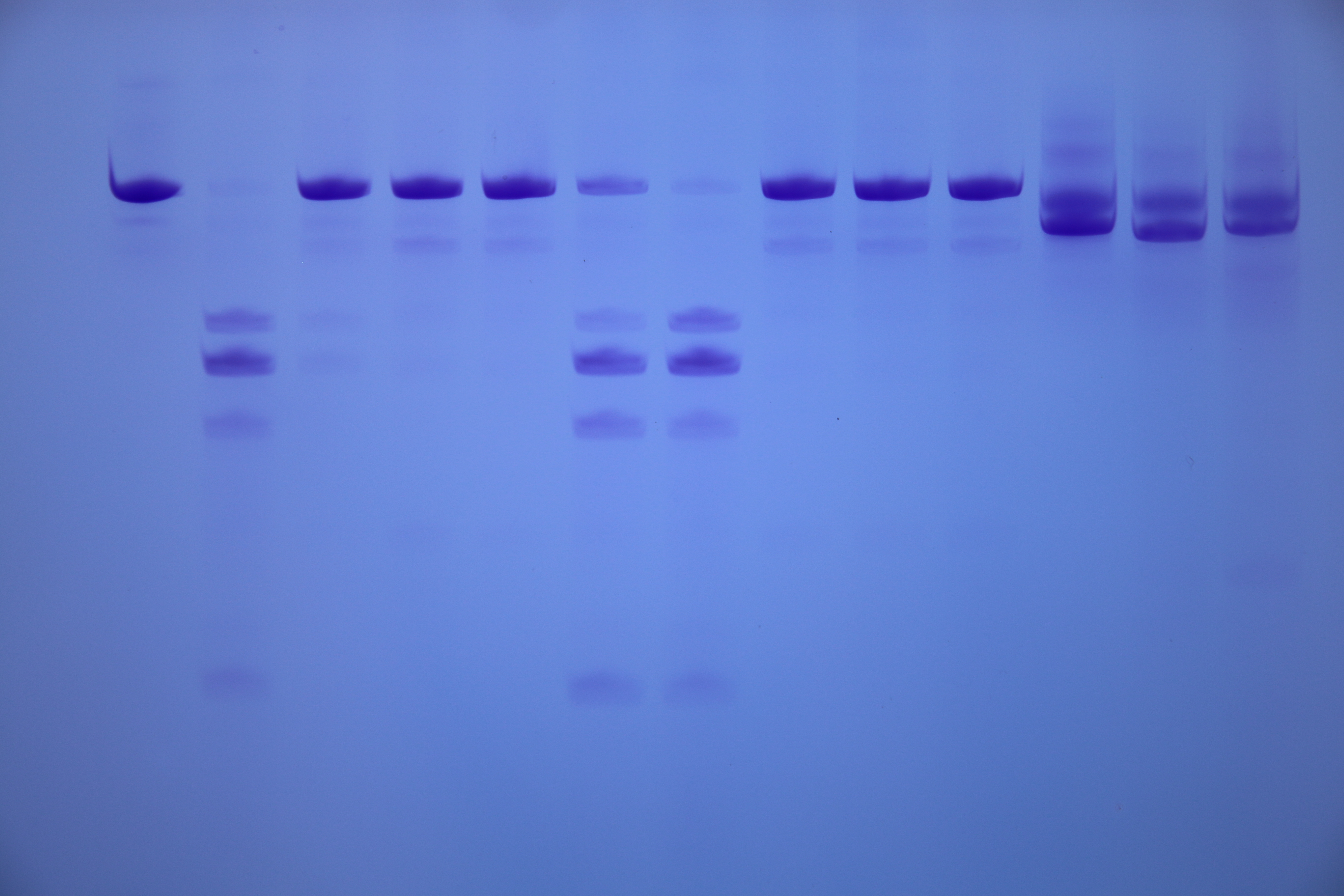

Supplement: Figure 5—source data 1. [file elife-86847-fig5-data1.zip › Figure 5-source data 1-1.JPG]

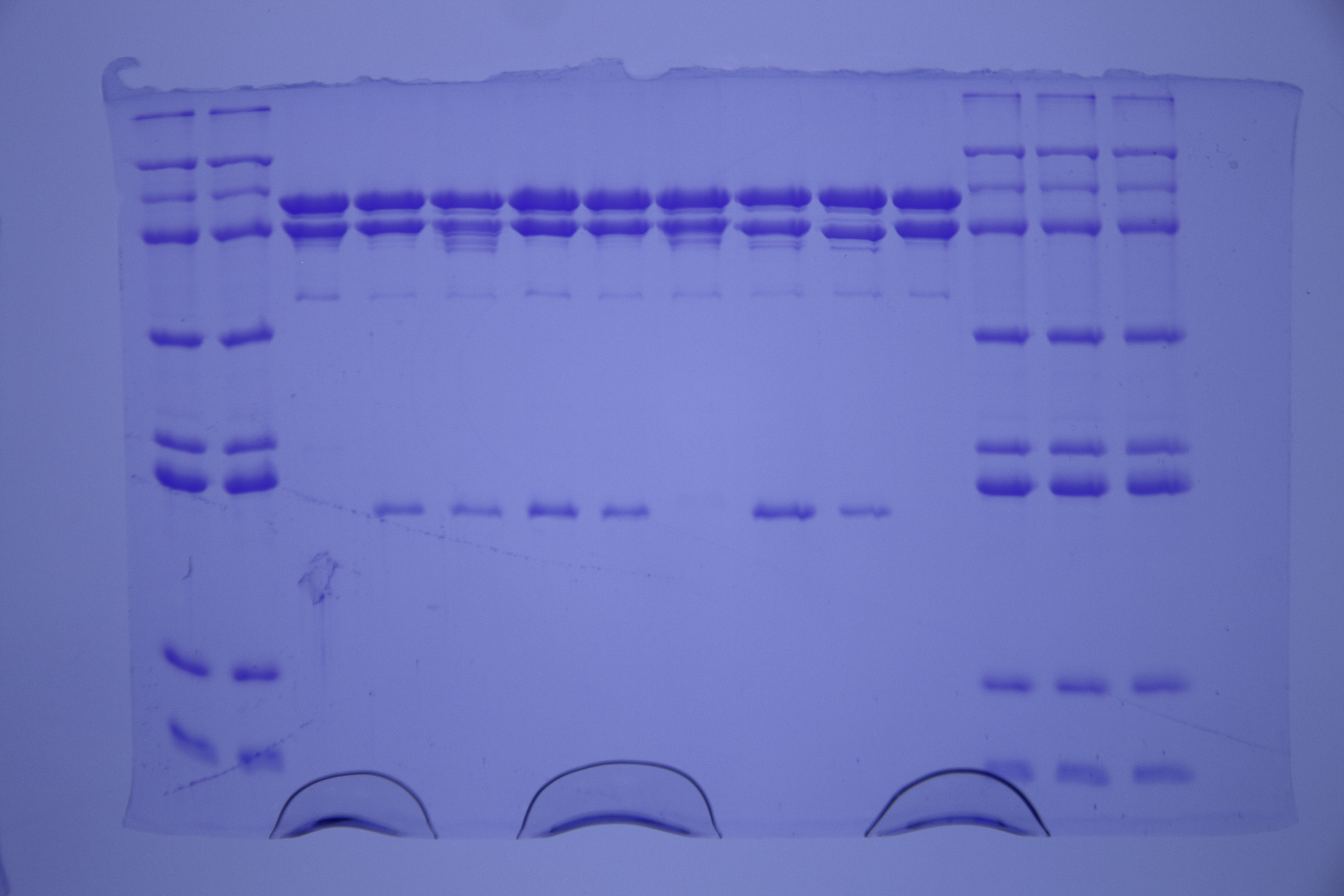

Supplement: Figure 5—source data 1. [file elife-86847-fig5-data1.zip › Figure 5-source data 1-2.JPG]

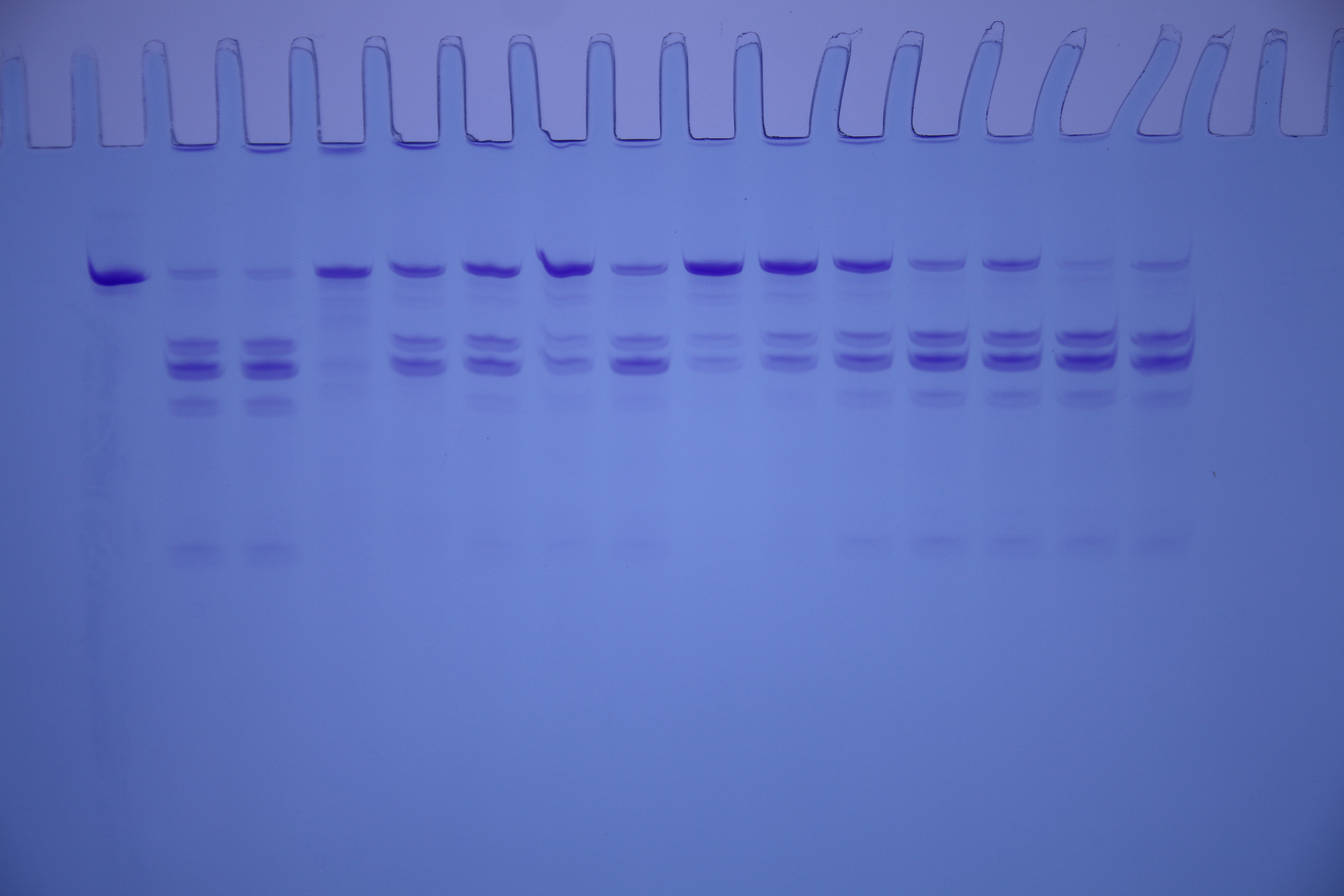

Supplement: Figure 5—source data 1. [file elife-86847-fig5-data1.zip › Figure 5-source data 1-3.JPG]

**Figure 5D**

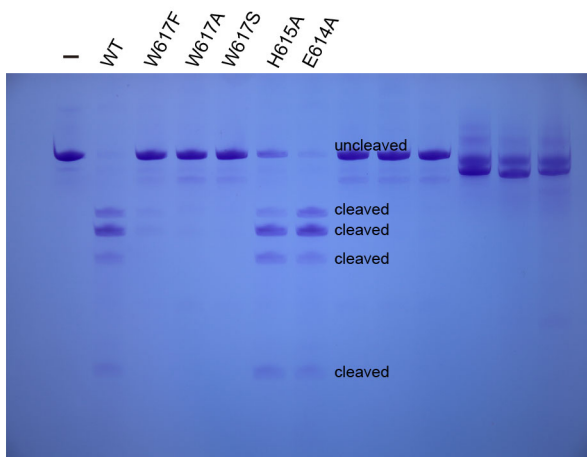

**Figure 5E**

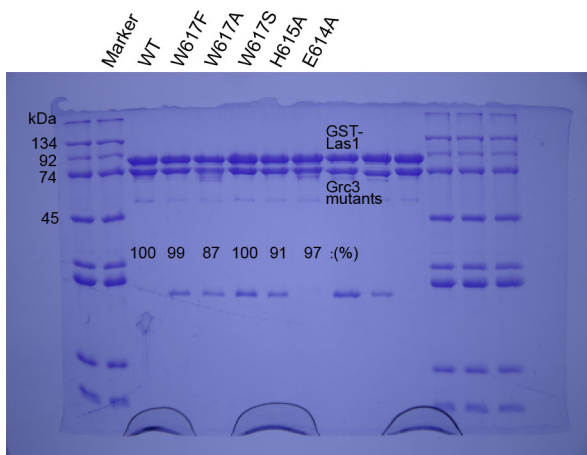

**Figure 5F**

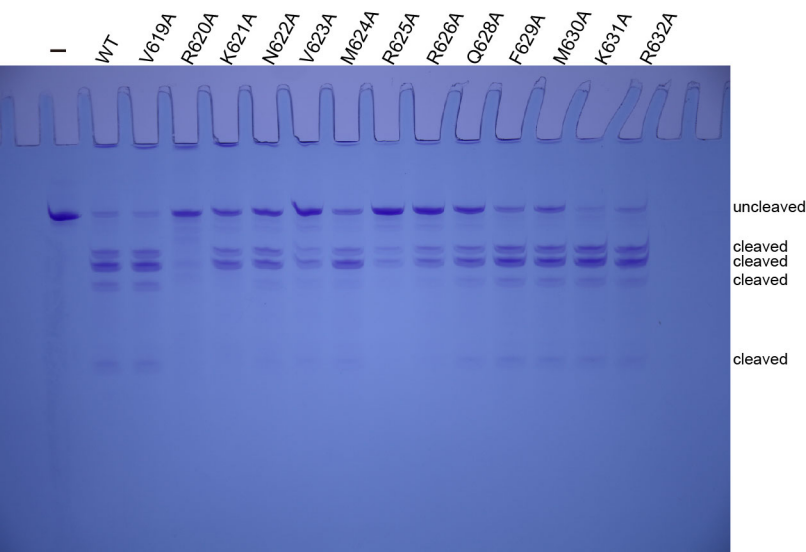

Supplement: Figure 5—source data 2. [file elife-86847-fig5-data2.zip › Figure 5-source data 2.pdf]

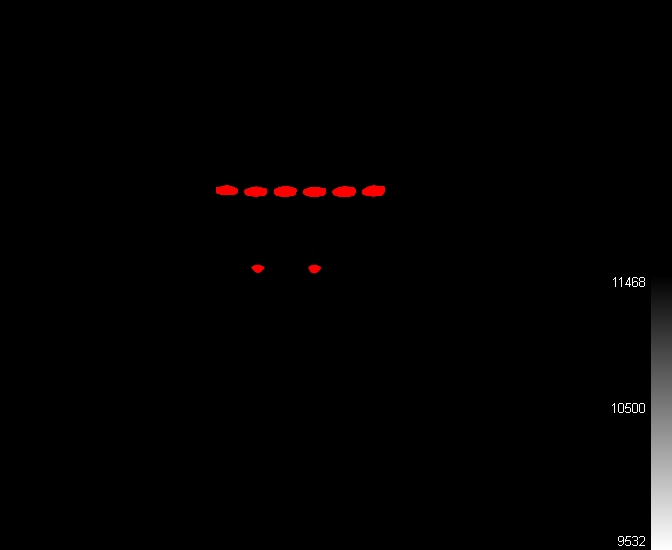

Supplement: Figure 5—figure supplement 4—source data 1. [file elife-86847-fig5-figsupp4-data1.zip › Figure 5-figure supplement 4-source data 1-1.jpg]

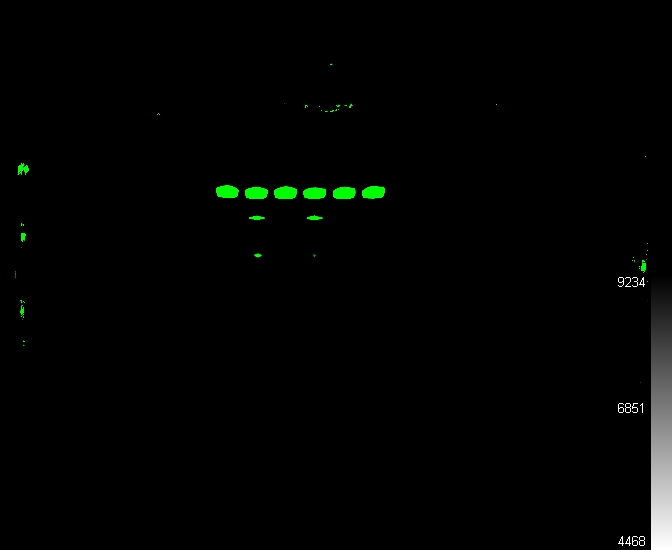

Supplement: Figure 5—figure supplement 4—source data 1. [file elife-86847-fig5-figsupp4-data1.zip › Figure 5-figure supplement 4-source data 1-2.jpg]

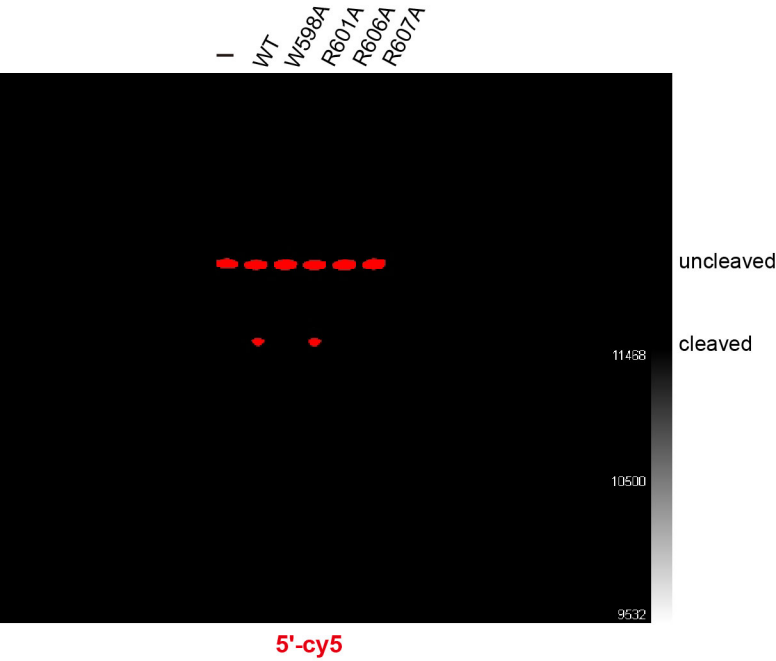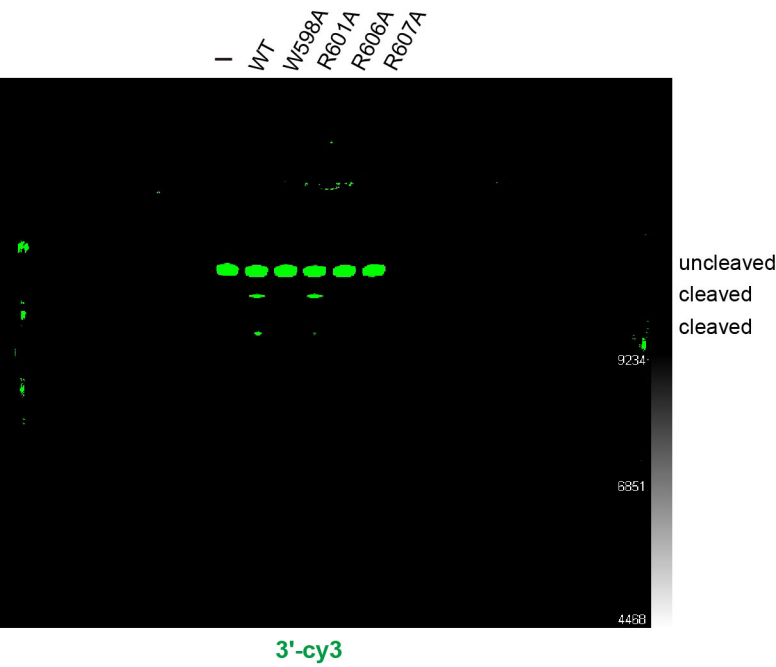

Supplement: Figure 5—figure supplement 4—source data 2. [file elife-86847-fig5-figsupp4-data2.zip › Figure 5-figure supplement 4-source data 2.pdf]

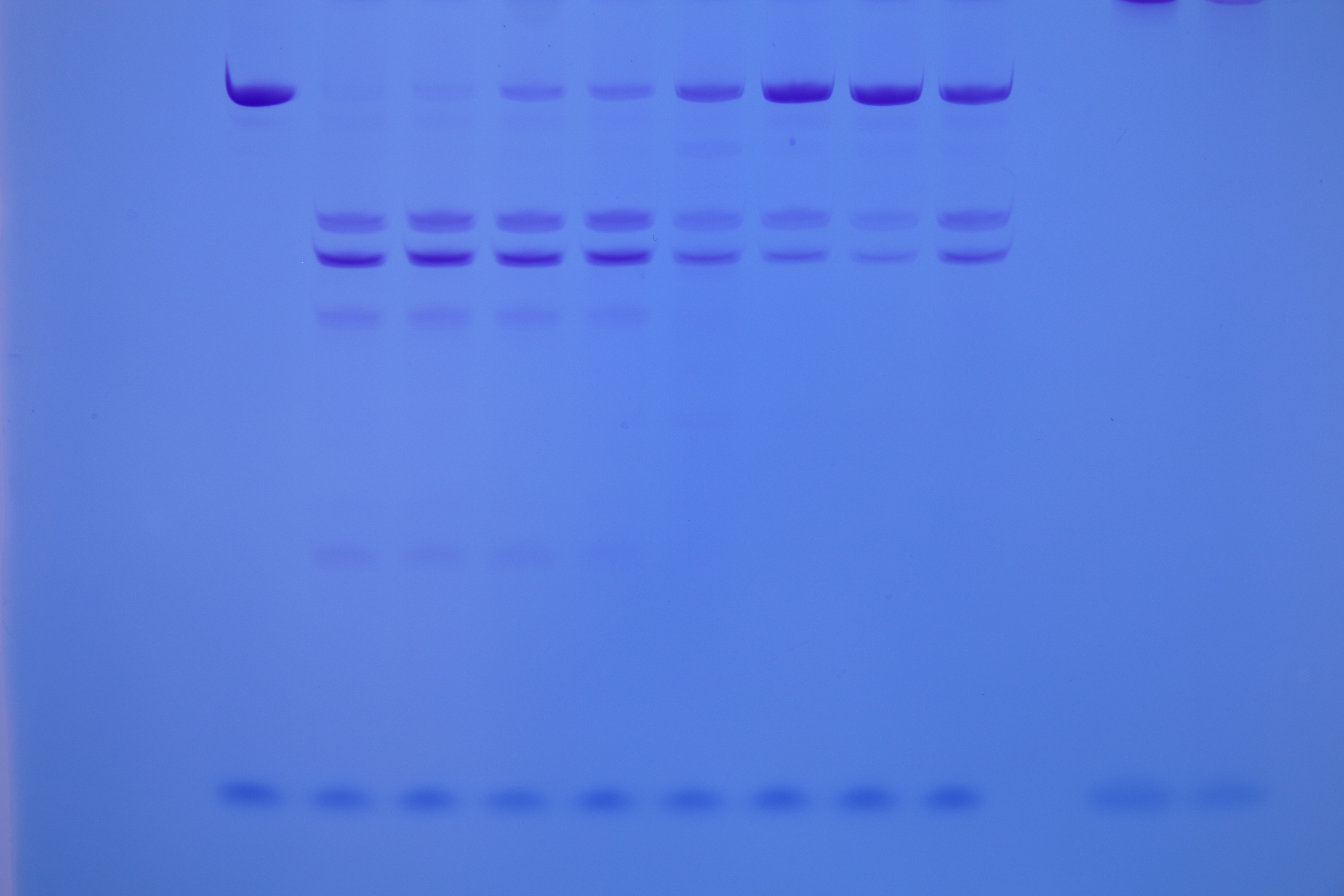

Supplement: Figure 6—figure supplement 2—source data 1. [file elife-86847-fig6-figsupp2-data1.zip › Figure 6-figure supplement 2-source data 1.JPG]

— WT N487A W488A W494A F499A Las1(1-497) Las1(1-492) Las1(1-484)

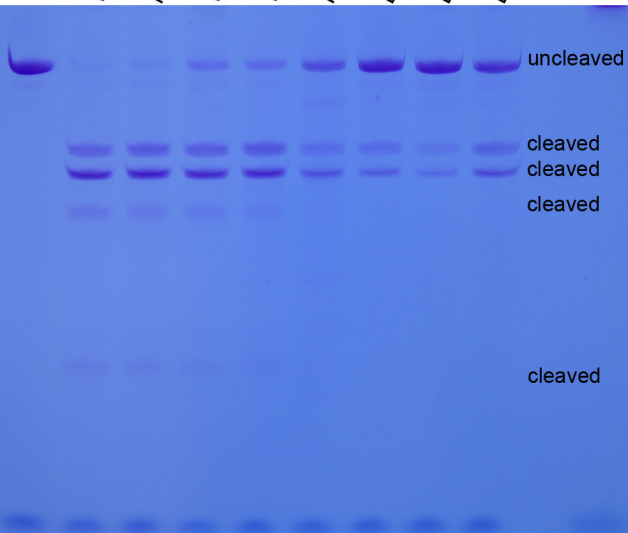

Supplement: Figure 6—figure supplement 2—source data 2. [file elife-86847-fig6-figsupp2-data2.zip › Figure 6-figure supplement 2-source data 2.pdf]

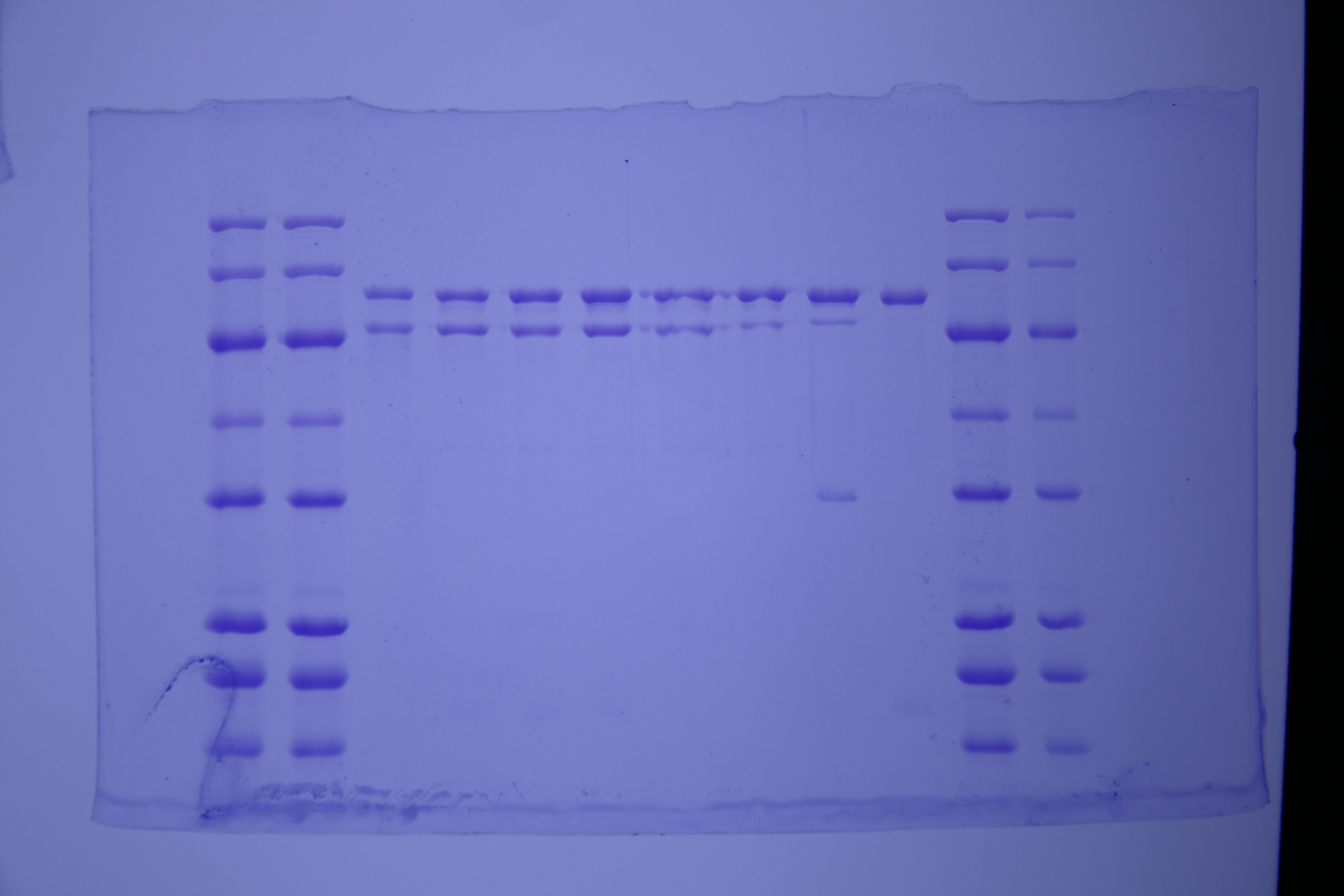

Supplement: Figure 6—figure supplement 3—source data 1. [file elife-86847-fig6-figsupp3-data1.zip › Figure 6-figure supplement 3-source data 1.JPG]

kDa

134

92

72

50

Marker

WT

N487A

W488A

W494A

F499A

Las1(1-497)

Las1(1-492)

Las1(1-484)

GST-Las1  
mutants

Grc3

100 100 100 100 80 22 20 0 : (%)

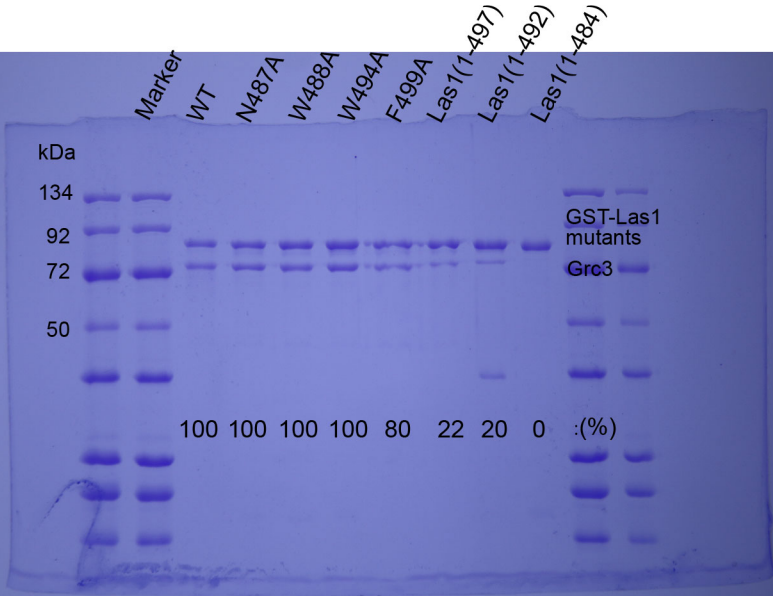

Supplement: Figure 6—figure supplement 3—source data 2. [file elife-86847-fig6-figsupp3-data2.zip › Figure 6-figure supplement 3-source data 2.pdf]
